# Detailed measurements of oesophageal pressure during mechanical ventilation with an advanced high-resolution manometry catheter

**DOI:** 10.1186/s13054-019-2484-8

**Published:** 2019-06-13

**Authors:** Per Persson, Rebecca Ahlstrand, Magni Gudmundsson, Alex de Leon, Stefan Lundin

**Affiliations:** 1 Department of Anaesthesiology and Intensive Care, Sahlgrenska University Hospital, Gothenburg, Sweden; 2 Department of Anaesthesia and Intensive Care, Örebro University Hospital, Örebro, Sweden

**Keywords:** Oesophagus (D004947), Mechanical ventilation (D012121), Respiratory mechanics (D015656), Manometry (D008365), Oesophageal pressure, Positive end-expiratory pressure

## Abstract

**Background:**

Oesophageal pressure (PES) is used for calculation of lung and chest wall mechanics and transpulmonary pressure during mechanical ventilation. Measurements performed with a balloon catheter are suggested as a basis for setting the ventilator; however, measurements are affected by several factors. High-resolution manometry (HRM) simultaneously measures pressures at every centimetre in the whole oesophagus and thereby provides extended information about oesophageal pressure. The aim of the present study was to evaluate the factors affecting oesophageal pressure using HRM.

**Methods:**

Oesophageal pressure was measured using a high-resolution manometry catheter in 20 mechanically ventilated patients (15 in the ICU and 5 in the OR). Different PEEP levels and different sizes of tidal volume were applied while pressures were measured continuously. In 10 patients, oesophageal pressure was also measured using a conventional balloon catheter for comparison. A retrospective analysis of oesophageal pressure measured with HRM in supine and sitting positions in 17 awake spontaneously breathing patients is also included.

**Results:**

HRM showed large variations in end-expiratory PES (PESEE) and tidal changes in PES (ΔPES) along the oesophagus. Mean intra-individual difference between the minimum and maximum end-expiratory oesophageal pressure (PESEE at baseline PEEP) and tidal variations in oesophageal pressure (ΔPES at tidal volume 6 ml/kg) recorded by HRM in the different sections of the oesophagus was 23.7 (7.9) cmH_2_O and 7.6 (3.9) cmH_2_O respectively. Oesophageal pressures were affected by tidal volume, level of PEEP, part of the oesophagus included and patient positioning. HRM identified simultaneous increases and decreases in PES within a majority of individual patients. Compared to sitting position, supine position increased PESEE (mean difference 12.3 cmH_2_O), pressure variation within individual patients and cardiac artefacts. The pressure measured with a balloon catheter did not correspond to the average pressure measured with HRM within the same part of the oesophagus.

**Conclusions:**

The intra-individual variability in PESEE and ΔPES is substantial, and as a result, the balloon on the conventional catheter is affected by many different pressures along its length. Oesophageal pressures are not only affected by lung and chest wall mechanics but are a complex product of many factors, which is not obvious during conventional measurements. For correct calculations of transpulmonary pressure, factors influencing oesophageal pressures need to be known. HRM, which is available at many hospitals, can be used to increase the knowledge concerning these factors.

**Trial registration:**

ClinicalTrials.gov, NCT02901158

**Electronic supplementary material:**

The online version of this article (10.1186/s13054-019-2484-8) contains supplementary material, which is available to authorized users.

## Background

Calculations of transpulmonary pressure during mechanical ventilation, performed in order to optimize ventilator settings, are dependent on oesophageal pressure measurements. Introduced already in 1878 [1], oesophageal pressure measurements became more widely used in respiratory mechanics research in the 1950s after the introduction of the balloon catheter [2–4]. Used as a surrogate of pleural pressure, the oesophageal pressure allows separation of lung and chest wall mechanics [5]. During the last decades, there has been an increasing interest in the technique due to the evolving knowledge about ventilator-induced lung injury which stresses the importance of lung protective ventilation and improved respiratory monitoring. Transpulmonary pressure calculated from oesophageal and airway pressures has previously been used to guide mechanical ventilation with promising results [6, 7], but the measurement of oesophageal pressure is complicated. Apart from the discussion of how to use the absolute or tidal changes in oesophageal pressures in calculations of transpulmonary pressure [8–10], there are several factors that influence the measurements. Both absolute end-expiratory and tidal variations in oesophageal pressures are affected by the positioning of the balloon [11, 12], filling volumes of the balloon [12, 13] and type of catheter used [14]. Due to the influence on the measurements of patient positioning [15–18], corrections of absolute values have previously been used [19, 20] although not in the study by Talmor et al. [6], who advocated the use of absolute oesophageal pressures. In a recent study, the absolute end-expiratory oesophageal pressure, uncorrected for a supine position and measured with a balloon catheter, seems to correspond to pleural pressure in the mid-lung region in lung-injured pigs and human cadavers [21]. Still, a better understanding of oesophageal pressures and influencing factors would be of great importance since measured values could have a profound effect on ventilator settings in the ICU and during general anaesthesia.

High-resolution manometry (HRM) has since its introduction in the 1990s developed into a common technique for oesophageal pressure measurements in gastroenterology [22]. The development of solid-state catheters has made it possible to simultaneously measure the pressure in the whole oesophagus using 36 pressure channels spaced 1 cm apart. HRM with solid-state catheters avoids many of the uncertainties associated with balloon catheters and introduces a possibility to validate the standard technique for oesophageal pressure measurements during mechanical ventilation. A tip manometry catheter has previously been used in oesophageal pressure measurements [23], but to our knowledge, the technology with multiple sensors has not previously been used for oesophageal pressure measurements in respiratory research.

The aim of this study was to use the HRM to evaluate oesophageal pressures during mechanical ventilation, to study the factors influencing oesophageal pressure such as body positioning and to make a comparison with a conventional balloon catheter regarding the degree of details and the amount of information obtained.

## Methods

### Patients

The study, registered in ClinicalTrials.gov (NCT02901158), was conducted at the Sahlgrenska University Hospital, Gothenburg, Sweden, after the approval from the Swedish Regional Ethics Committee (mechanically ventilated patients: Ref 615-13; spontaneously breathing patients: Ref T1040-16). Informed written consent was obtained from patients or next of kin before inclusion. Measurements were performed during controlled mechanical ventilation in 15 patients with respiratory failure in the ICU and in 5 lung healthy patients in the operating room during general anaesthesia prior to the start of surgery. Measurements previously performed at the gastroenterology department in 17 lung-healthy, awake and spontaneously breathing patients in sitting and supine positions were also included in the study.

### Measurements

Conventional measurement of oesophageal pressures in mechanically ventilated patients was performed using a balloon catheter (Nutrivent™, SIDAM, Mirandola, Italy) connected to a standard pressure transducer (DTXplus, Argon Medical Devices Inc., Plano, TX, USA). The oesophageal balloon was positioned 40–42 cm from the nostril and inflated with 4 ml of air according to the instruction from the manufacturer. Correct positioning was tested with a “positive pressure occlusion test” [11]. Detailed measurements of oesophageal pressures were performed using high-resolution solid-state oesophageal manometry (HRM) catheter (Sierra Scientific Instruments, Inc., Los Angeles, CA, USA) with 36 pressure channels spaced 1 cm apart, each consisting of 12 circumferential pressure sensors (in total 432 pressure sensors). The large number of sensors allows separate measurements of pressure all the way from the pharynx to the stomach. The HRM catheter is constructed for 25O separate measurements/catheter (cost approx. 50 €/measurement procedure). In mechanically ventilated patients, airway pressures and tidal volumes were measured by the Servo-I ventilator or Flow-I anaesthesia machine (Maquet Critical Care, Solna, Sweden) and presented by a Maquet dedicated software. Change in end-expiratory lung volume following a change of PEEP was calculated as the cumulative differences between inspiratory and expiratory tidal volumes during 15 breaths [24].

### Analysis

Data from the HRM were analysed in the ManoView software (Sierra Scientific Instruments, Inc. Los Angeles, CA, USA). In mechanically ventilated patients, pressures from sensors 2 cm apart along a length of 22 cm were included in the analysis (Additional file 1: Figure S1). The most caudal sensor with typical tidal oesophageal swings seen at any PEEP level was set as the lowest level, with a mean distance of 48 cm from the nostril. At the end of each measurement procedure, pressure drift was determined and compensated for before analysis according to the recommendations from the manufacturer. In two patients, pressures from three sensors were replaced by an average of the pressures from sensors 1 cm cranially and 1 cm caudally because of sensor dysfunction. One patient was excluded from the third PEEP step/tidal volume because of high airway pressures. In spontaneously breathing patients, pressures from sensors 2 cm apart between 24 and 46 cm from the nostril during time periods without visible oesophageal muscle activities were included in the analyses. In three spontaneously breathing patients, pressures from the lowest (one patient) or the two lowest (two patients) levels were excluded from the analyses due to the large pressure variations caused by oesophageal muscle activity.

Pressure from 22 cm of the oesophagus, ESO_TOT_ (12 levels, 2 cm apart), and pressures between 30 and 42 cm from the nostril, ESO_LOW_ (6–7 levels, 2 cm apart), were analysed separately. The oesophagus between 30 and 42 cm from the nostril is the recommended position for the 10-cm-long oesophageal balloon (Nutrivent™, SIDAM, Mirandola, Italy). This position is also used in several previous studies [6, 17, 18] and is likely unaffected by high pressures in the lower oesophageal sphincter.

### Statistical analysis

All data are presented as mean with standard deviation (SD) and median (min-max) for continuous variables and as *n* (%) for categorical values. From the measurements with HRM, the mean end-expiratory oesophageal pressure and mean tidal variation in oesophageal pressures in a patient were calculated (average pressure) as were the standard deviations within a patient (within-subject standard deviation). Coefficient of variation is calculated as the standard deviation divided by the mean (SD/mean). Significance of the differences are analysed by calculation of the 95% confidence interval (CI) of the mean difference and interpreted as significant if the confidence interval is separated from zero.

### Protocol

Mechanically ventilated patients were under deep sedation and given rocuronium to achieve muscle relaxation during measurements. End-inspiratory and end-expiratory oesophageal pressures were recorded at four or five different PEEP levels (see below) with a tidal volume of approximately 6 ml/kg ideal body weight (IBW). Baseline PEEP level was decided by the clinician. Three different PEEP steps were performed from baseline aiming at an increase of end-expiratory lung volume of approximately 1, 1.5 and 2 times the tidal volume. After PEEP was returned to baseline, the tidal volume was set equal to the measured change in end-expiratory lung volume. During these three different sizes of tidal volumes (VT 1, VT 2 and VT 3), tidal changes in oesophageal pressure (ΔPES) were measured. At the end of the protocol, PEEP was changed to zero (ZEEP) if considered safe for the patient (performed in 16 patients). Chest wall compressions (used in “positive pressure occlusions test”) were performed in 14 patients before ending the measurements with HRM catheter. In 10 patients, a balloon catheter was placed after the removal of the HRM catheter and end-inspiratory and end-expiratory oesophageal pressures were recorded at 2–3 of the previous PEEP levels with tidal volumes ≈ 6 ml/kg IBW.

In spontaneously breathing patients, the oesophageal pressure measurement with HRM was part of a routine examination in the gastroenterology policlinic due to gastro-oesophageal symptoms. In each patient, measurements were performed both in supine and sitting positions and examinations were analysed retrospectively.

## Results

### Mechanically ventilated patients

Patient mean (SD) age was 56 (16) years, BMI 26.5 (5.3) kg/m^2^ and PaO2/FiO2 ratio 250 (86) mmHg (range 80–350 mmHg). For patient characteristics, see Additional file 1: Table S1. During HRM measurements, the mean (SD) PEEP level at baseline was 8.1 (1.9) cmH_2_O and then changed to 13.2 (2.0), 15.3 (2.0), 17.0 (2.0) and 0.3 (0.3) cmH_2_O. The total PEEP-induced changes in end-expiratory lung volume during these PEEP steps were 441 (94) ml, 629 (126) ml, 858 (148) ml and 796 (414) ml respectively. Tidal volumes during ΔPES measurements were 451 (87) ml, 637 (119) and 863(146) ml.

#### End-expiratory oesophageal pressure

**Fig. 1:**
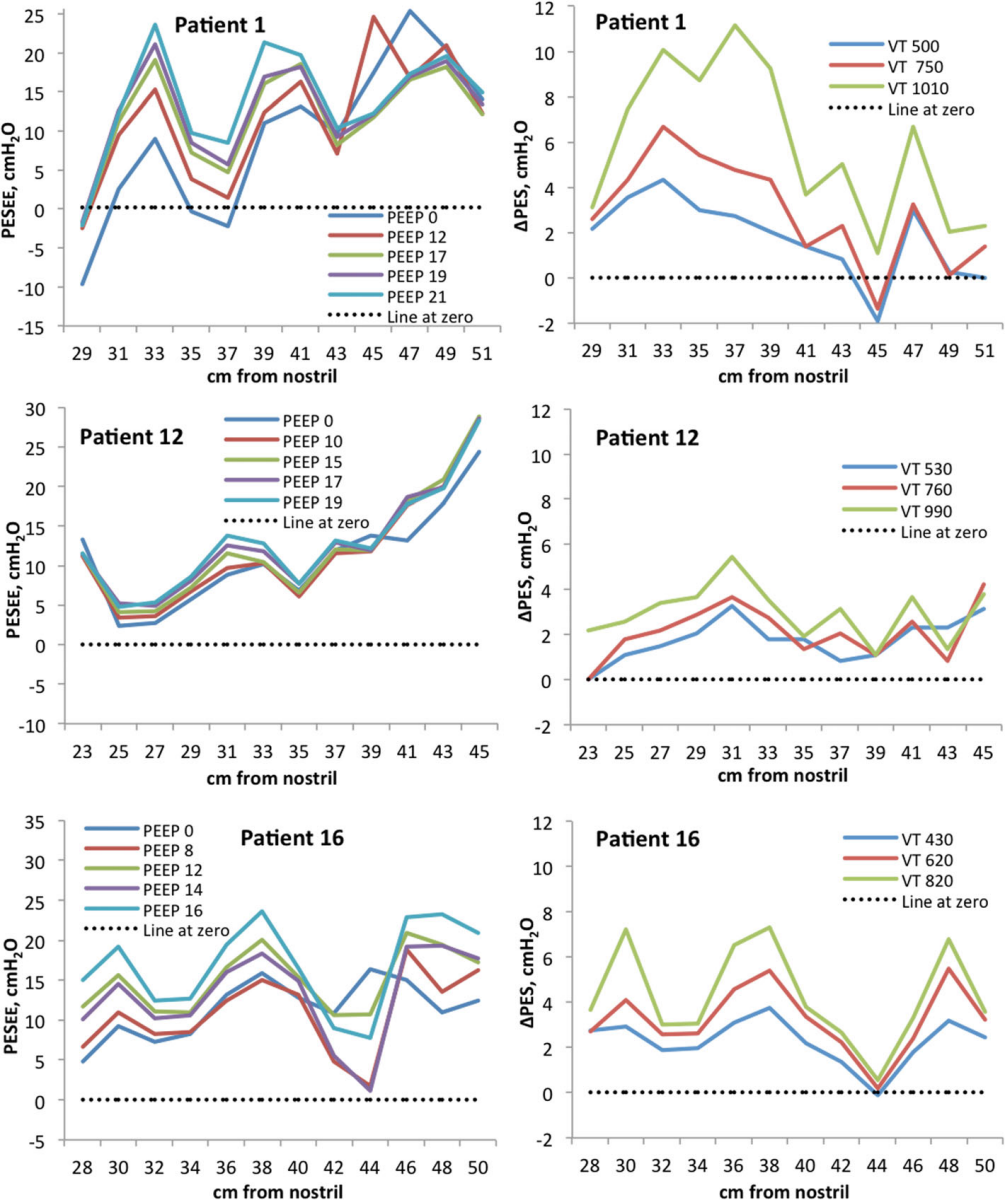
Left panel: end-expiratory oesophageal pressure (PESEE) along the oesophagus at different PEEP levels in three mechanically ventilated representative patients. Right panel: tidal variations in oesophageal pressure (ΔPES) along the oesophagus at different tidal volumes in three mechanically ventilated representative patients. For curves from all patients, see Additional file 1: Figures S2 and S6.

End-expiratory oesophageal pressures varied along the oesophagus. The mean within-subject SD was 4.7–7.2 cmH_2_O, and mean coefficient of variation 32–58% depending on the PEEP level and part of the oesophagus included in the analysis (Table 1, Fig. 1 and Additional file 1: Figure S2, Table S2). Mean intra-individual difference between the minimum and maximum end-expiratory oesophageal pressures recorded by HRM in the different sections of the oesophagus at baseline PEEP was 23.7 (7.9) cmH_2_O (ESO_TOT_) and 13.3 (8.2) cmH_2_O (ESO_LOW_). The pattern of pressure variation along the length of the oesophagus was different between the patients (Fig. 1 and Additional file 1: Figure S2). The average end-expiratory oesophageal pressure increased when PEEP was increased (Table 1, Fig. 1 and Additional file 1: Figure S2). An increase of PEEP causes a single-step increase in end-expiratory oesophageal pressure measured with the balloon catheter. Measurements with HRM revealed a variable effect on oesophageal pressures when PEEP was increased, and often both increases and decreases in end-expiratory oesophageal pressures were seen within the same patient (Fig. 1 and Additional file 1: Figures S2, S3, S4, S5).

**Table 1:** Oesophageal pressure measured with high-resolution manometry catheter

Mechanically ventilated patients						
Part of oesophagus included	End-expiratory oesophageal pressure					
	Average pressure			Within-subject standard deviation		
	Low PEEP^a^ (*n*=20) ≈ 9 cmH_2_O Mean (SD) Median (min; max)	High PEEP^b^ (*n*=20) ≈ 17 cmH_2_O Mean (SD) Median (min; max)	Difference High-low Mean (SD) Median (min; max) 95% CI	Low PEEP^a^ (*n* = 20) ≈ 9 cmH_2_O Mean (SD) Median (min; max) 95% CI	High PEEP^b^ (*n* = 20) ≈ 17 cmH_2_O Mean (SD) Median (min; max) 95% CI	Difference High-low Mean (SD) Median (min; max) 95% CI
22 cm of the oesophagus (ESO_TOT_)	13.4 (3.7) 12.9 (6.4; 20.1)	16.7 (4.2) 16.0 (11.0; 26.0)	3.4 (2.1) 3.3 (− 0.56; 6.1) 2.4; 4.4*	7.2 (2.3) 7.3 (4.3; 12.6) 6.1; 8.3*	6.5 (2.2) 6.0 (2.7; 10.6) 5.4; 7.5*	− 0.8 (1.6) − 0.8 (− 3.5; 2.2) − 1.5; − 0.04*
30–42 cm from the nostril (ESO_LOW_)	11.8 (5.2) 11.6 (3.1; 23.5)	15.4 (5.2) 15.9 (7.4; 27.5)	3.6 (2.3) 3.8 (− 1.1; 7.6) 2.6; 4.7*	5.0 (2.9) 4.2 (1.8; 15.1) 3.6; 6.3*	4.7 (2.9) 4.4 (1.3; 13.8) 3.4; 6.1*	− 0.23 (1.4) − 0.32 (− 2.6; 3.9) − 0.91; 0.44
Difference “22 cm” vs. “30–42 cm” Mean (SD) Median (min-max) 95% CI	1.6 (2.4) 1.7 (− 3.5; 5.9) 0.42; 2.7*	1.3 (2.2) 1.3 (− 2.9; 5.3) 0.28; 2.3*		2.3 (2.8) 2.1 (− 3.3; 7.2) 0.94; 3.6*	1.7 (2.6) 1.0 (− 3.1; 7.7) 0.50; 2.9*	
Part of oesophagus included	Tidal change in oesophageal pressure					
	Average pressure			Within-subject standard deviation		
	VT 1^c^ (*n* = 20) ≈ 6 ml/kg Mean (SD) Median (min; max)	VT 3^d^ (*n* = 19) ≈ 12 ml/kg Mean (SD) Median (min; max)	Difference VT 3–VT 1 Mean (SD) Median (min; max) 95% CI	VT1^c^ (*n* = 20) ≈ 6 ml/kg Mean (SD) Median (min; max) 95% CI	VT 3^d^ (*n* = 19) ≈ 12 ml/kg Mean (SD) Median (min; max) 95% CI	Difference VT 3–VT 1 Mean (SD) Median (min; max) 95% CI
22 cm of oesophagus (ESO_TOT_)	2.4 (1.3) 2.3 (0.2; 5.7)	4.1 (1.6) 4.3 (0.7; 6.6)	1.8 (0.92) 1.9 (0.36; 4.1) 1.4; 2.2*	2.2 (1.0) 2.0 (0.8; 4.4) 1.7; 2.6*	3.7 (1.6) 3.9 (1.2; 6.2) 2.9; 4.5*	1.5 (1.1) 1.1 (− 0.24; 4.1) 0.94; 2.0*
30–42 cm from the nostril (ESO_LOW_)	2.5 (1.9) 2.5 (− 2.1; 7.3)	4.1 (2.3) 4.4 (− 1.0; 8.4)	1.7 (1.4) 1.9 (− 1.1; 5.6) 1.0; 2.3*	2.0 (1.2) 1.7 (0.8; 4.9) 1.4; 2.5*	4.5 (1.0) 4.4 (2.3; 6.6) 4.0; 4.9*	2.5 (1.3) 2.7 (− 0.48; 4.7) 1.9; 3.1*
Difference “22 cm” vs. “30–42 cm” Mean (SD) Median (min; max) 95% CI	− 0.12 (0.87) − 0.08 (− 1.6; 2.6) − 0.53; 0.29	0.01 (1.2) − 0.16 (− 2.5; 3.7) − 0.60; 0.59		0.20 (0.64) 0.19 (− 1.1; 1.4) − 0.10; 0.51	− 0.81 (1.8) − 0.53 (− 3.3; 2.9) − 1.7; 0.04	
Spontaneously breathing patients						
Part of oesophagus included	End-expiratory oesophageal pressure					
	Average pressure			Within-subject standard deviation		
	Sitting (*n* = 17) Mean (SD) Median (min; max)	Supine (*n* = 17) Mean (SD) Median (min; max)	Difference Supine vs. sitting Mean (SD) Median (min; max) 95% CI	Sitting (*n* = 17) Mean (SD) Median (min; max) 95% CI	Supine (*n* = 17) Mean (SD) Median (min; max) 95% CI	Difference Supine vs. sitting Mean (SD) Median (min; max) 95% CI
24–46 cm from the nostril^e^ (ESO_TOT_)	3.3 (6.2) 1.7 (− 4.5; 21.2)	15.6 (7.2) 15.6 (− 0.5; 28.1)	12.3 (7.1) 10.3 (2.2; 25.7) 8.6; 15.9*	5.4 (2.2) 5.4 (2.0; 8.9) 4.2; 6.5*	7.5 (3.5) 6.7 (1.7; 13.8) 5.7; 9.4*	2.2 (3.5) 2.3 (− 4.2; 8.0) 0.37; 4.0*
30–42 cm from the nostril (ESO_LOW_)	2.8 (7.0) 1.4 (− 5.0; 24.8)	16.3 (8.3) 16.3 (− 0.2; 29.8)	13.5 (8.5) 11.9 (− 1.0; 29.9) 9.2; 17.9*	3.4 (1.9) 3.2 (0.6; 8.0) 2.4; 4.3*	5.7 (3.0) 5.2 (1.5; 12.8) 4.2; 7.3*	2.4 (3.6) 2.3 (− 3.8; 11.2) 0.48; 4.2*
Difference “24–46 cm” vs. “30–42 cm” Mean (SD) Median (min-max) 95% CI	0.52 (1.6) 0.59 (− 3.6; 2.4) − 0.31; 1.3	− 0.76 (2.4) − 1.1 (− 4.7; 4.8) − 2.0; 0.49		2.0 (1.6) 1.4 (− 0.04; 5.7) 1.2; 2.8*	1.8 (2.5) 1.0 (− 1.2; 6.5) 0.54; 3.1*	
Part of the oesophagus included	Tidal change in oesophageal pressure					
	Average change in pressure			Within-subject standard deviation		
	Sitting (*n* = 17) Mean (SD) Median (min; max)	Supine (*n* = 17) Mean (SD) Median (min; max)	Difference Supine vs. sitting Mean (SD) Median (min; max) 95% CI	Sitting (*n* = 17) Mean (SD) Median (min; max) 95% CI	Supine (*n* = 17) Mean (SD) Median (min; max) 95% CI	Differences Supine vs. sitting Mean (SD) Median (min; max) 95% CI
24–46 cm from the nostril^e^ (ESO_TOT_)	6.3 (2.4) 5.6 (2.3; 10.6)	4.9 (3.1) 4.3 (− 1.2; 9.6)	− 1.4 (4.0) − 1.0 (− 7.1; 4.7) − 3.5; 0.63	4.9 (4.8) 2.7 (0.7; 18.1) 2.4; 7.4*	4.8 (2.6) 4.5 (0.4; 9.6) 3.4; 6.1*	− 0.13 (3.8) 0.76 (− 12.2; 4.3) − 2.1; 1.8
30–42 cm from the nostril (ESO_LOW_)	7.9 (3.2) 7.9 (2.7–13.2)	5.0 (3.3) 5.2 (− 2.2; 10.7)	− 2.8 (4.6) − 1.6 (− 11.2; 4.2) − 5.2; − 0.46*	2.0 (2.3) 1.2 (0.3; 7.8) 0.9; 3.2*	3.7 (3.2) 2.8 (0.4; 9.3) 2,1; 5,3*	1.7 (4.0) 1.4 (− 7.3; 7.9) − 0.37; 3.7
Difference “24–46 cm” vs. “30–42 cm” Mean (SD) Median (min-max) 95% CI	− 1.5 (2.0) − 0.54 (− 5.7; 1.0) − 2.6; − 0.51*	− 0.16 (1.5) − 0.06 (− 3.1; 2.4) − 0.92; 0.60		2.9 (4.7) 1.3 (− 0.53; 17.1) 0.49; 5.3*	1.1 (2.0) 0.88 (− 2.1; 5.1) 0.08; 2.1*	

*Confidence interval separated from 0

^a^Low PEEP (PEEP closest to 10 cmH_2_O, mean 9.2 (1.4) cmH_2_O)

^b^High PEEP (Highest PEEP, mean 17.0 (2.0) cmH_2_O)

^c^VT 1 = tidal volumes 6.5 (1.2) ml/kg IBW

^d^VT 3 = tidal volumes 12.0 (3.6) ml/kg IBW

^e^Proximal and distal high pressures (> 50 cmH_2_O) excluded, see text

#### Tidal oesophageal pressure variations

**Fig. 2:**
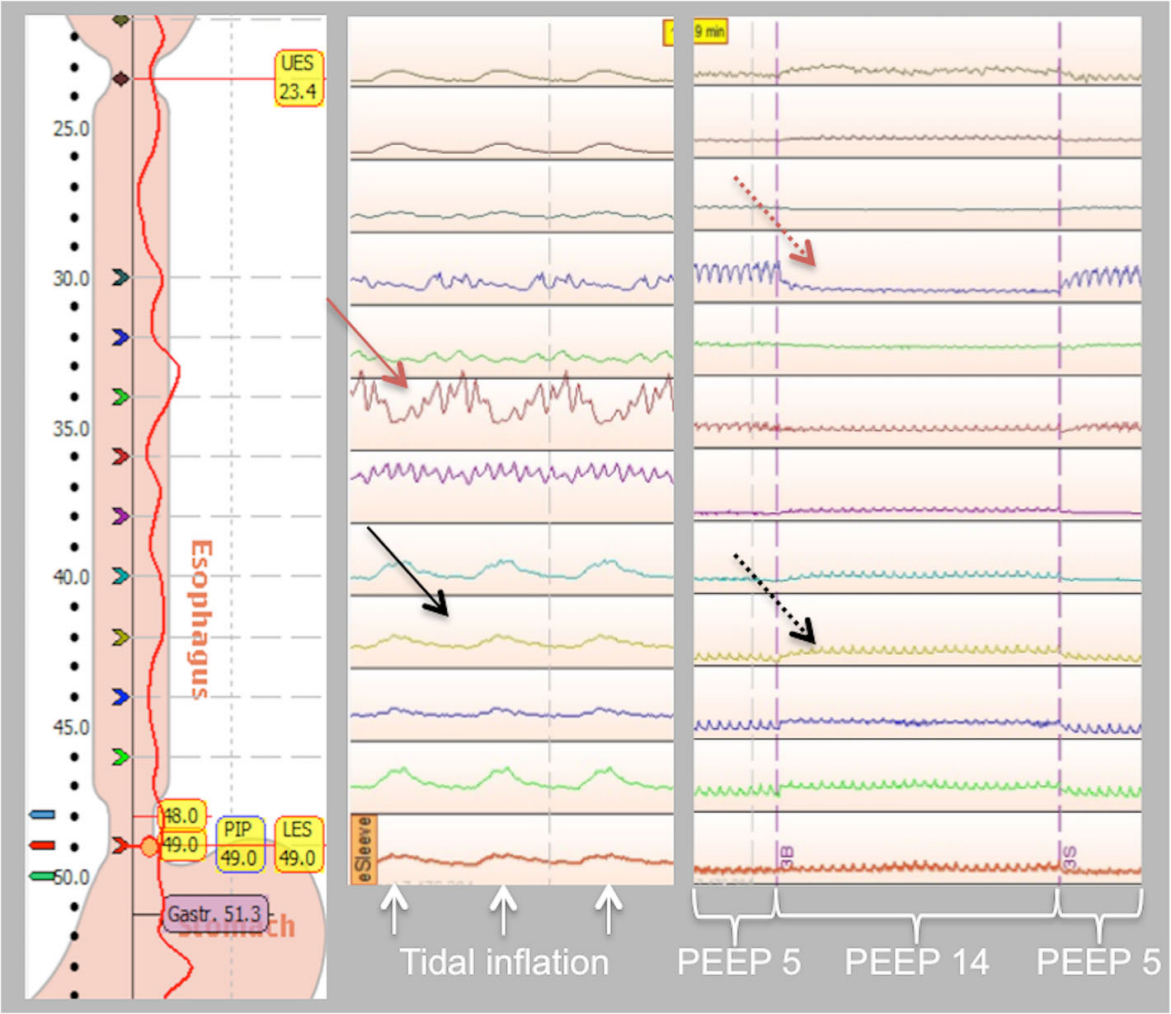
Left panel: picture from one patient in ManoView showing the oesophagus with a red pressure curve created from pressure recording 1 cm apart along its full length. Numbers on the left side represents cm from the nostril. UES, upper oesophageal sphincter; LES, lower oesophageal sphincter. Middle panel: within a patient both positive (black arrow) and negative (red arrow) ΔPES may be seen during tidal inflation. Pressure curve recorded 2 cm apart 26–48 cm from the nostril. Right panel: both increases (black dotted arrow) and decreases (red dotted arrow) in end-expiratory oesophageal pressure may be seen when PEEP is increased. Pressure curves recorded 2 cm apart 27–49 cm from the nostril. Pressure recordings from representative patients

Tidal changes in oesophageal pressure (ΔPES) varied along the oesophagus. The within-subject SD was 2.0–4.5 cmH_2_O and the mean coefficient of variation 83–151% depending on the size of tidal volume and part of the oesophagus included. The variation within a patient was higher at higher tidal volume (Table 1, Fig. 1 and Additional file 1: Table S2, Figure S6). Mean intra-individual difference between the minimum and maximum tidal variations in oesophageal pressure recorded by HRM in the different sections of the oesophagus was 7.6 (3.9) cmH_2_O (ESO_TOT_) or 5.5 (3.6) cmH2O (ESO_LOW_) during tidal inflation of 6 ml/kg IBW. During tidal inflation (VT ≈ 6 ml/kg IBW at baseline PEEP), both positive and negative ΔPES were seen in 12 of 20 patients (ESO_TOT_) and 10 of 20 patients (ESO_LOW_). For curves with ΔPES at different PEEP levels, see Additional file 1: Figure S7. ΔPES increased significantly when the tidal volume was increased, but locally within a patient, the effect varied. Within a patient, both increases and decreases in ΔPES could be seen when tidal volumes were increased (Figs. 1 and 2 and Additional file 1: Figure S6).

#### Comparison of high-resolution manometry with a balloon catheter

**Fig. 3:**
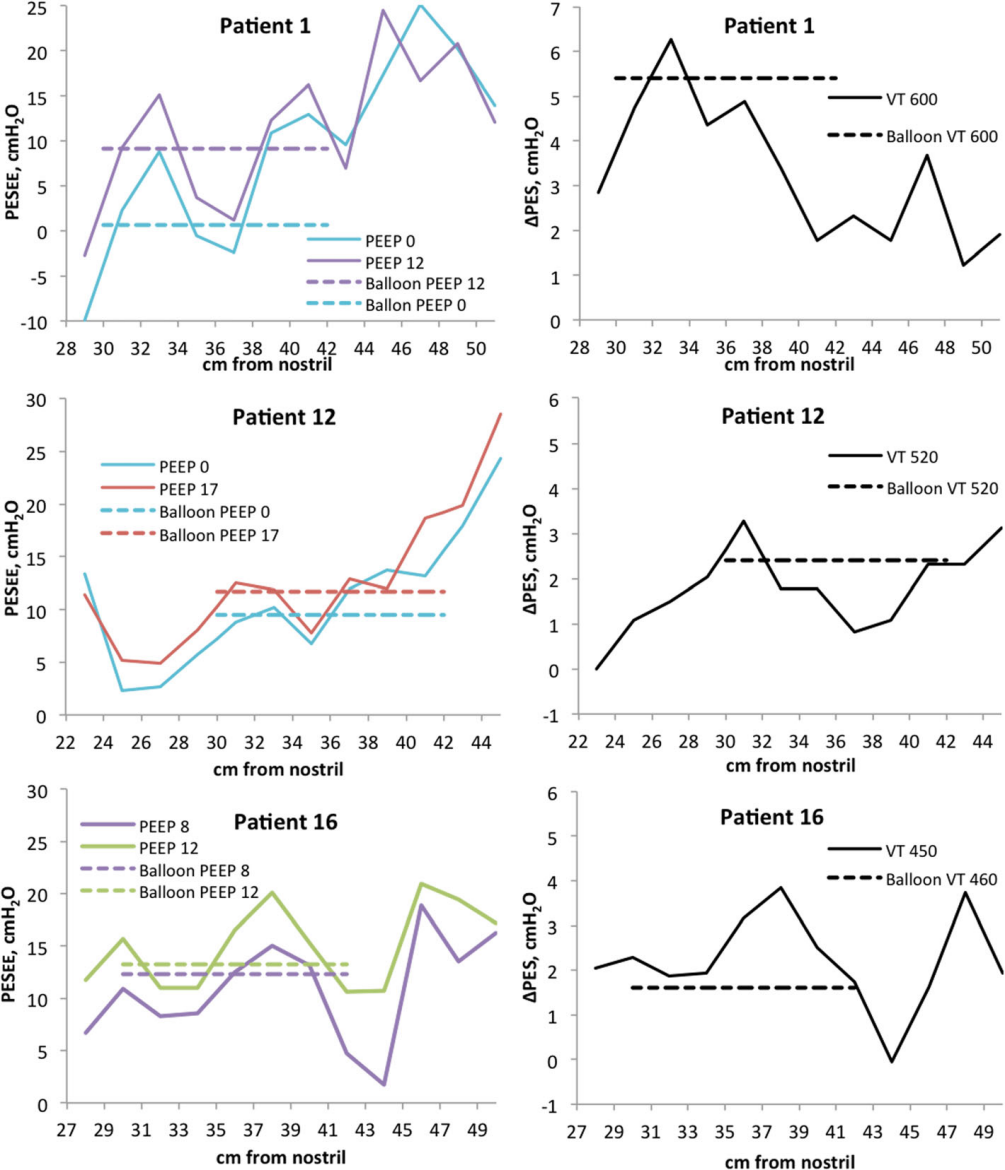
Comparison between the measurements of end-expiratory oesophageal pressure (left panel) and ΔPES (right panel) from HRM catheter and balloon catheter. Three representative patients. For all patients, see Additional file 1: Figures S8 and S9.

In contrast to the measurements from conventional balloon catheters, HRM reveals the described variation in pressures along the oesophagus and simultaneous increases and decreases in pressure locally within a patient during tidal inflation and increase of PEEP (Fig. 3 and Additional file 1: Figures S8-S9). The average end-expiratory pressure and average ΔPES from HRM measurements (ESO_LOW_) deviated significantly but not systematically from the pressures measured with the balloon catheter in the same part of the oesophagus (Additional file 1: Table S2 and Figure S10).

#### Oesophageal pressure during chest wall compression

During chest wall compression, the mean average changes in oesophageal pressure measured with HRM were 10.8 (5.5) cmH_2_O. The mean within-patient SD was 4.1 (1.8) cmH_2_O (ESO_TOT_) or 2.9 (1.6) cmH_2_O (ESO_LOW_). The mean intra-individual difference between the largest and smallest changes in oesophageal pressure during chest wall compression was 14.6 (7.6) cmH_2_O (ESO_TOT_) or 8.3 (4.5) cmH_2_O (ESO_LOW_) (Additional file 1: Figure S11).

### Spontaneously breathing patients

**Fig. 4:**
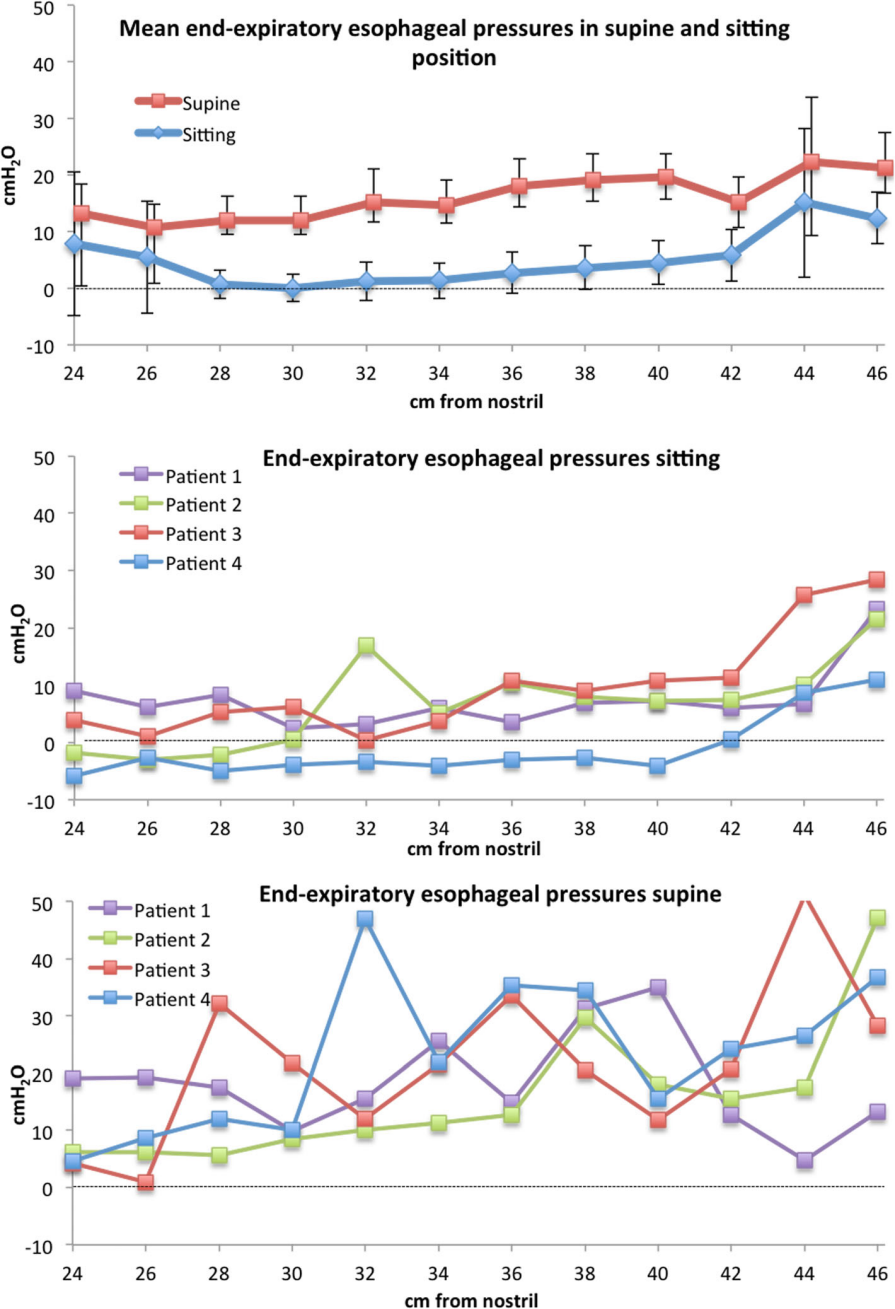
Top panel: mean end-expiratory oesophageal pressures in 17 spontaneously breathing patients in supine and sitting positions. All pressures included, see text. Bars show 95% confidence interval. Bottom two panels: end-expiratory pressures in four patients in supine and sitting positions. For curves from all patients, see Additional file 1: Figure S11

The average end-expiratory oesophageal pressure was significantly higher in supine compared to sitting position, mean difference of 12.3 cmH_2_O, 95% CI 8.6–15.9 (ESO_TOT_). Isolated high pressures (> 50 cmH_2_O) in the most proximal and distal part of the oesophagus in patients 5, 6, 15 and 16 (Fig. 4 and Additional file 1: Figure S12) were interpreted as due to the upper and lower oesophageal sphincter and excluded from the calculations. For end-expiratory oesophageal pressure, the within-subject SD was significantly higher in supine compared to sitting position, mean difference 2.2 cmH_2_O (95% CI 0.4–4.0 cmH_2_O) (Table 1, Fig. 4 and Additional file 1: Figure S12). In a supine position, cardiac oscillations are more prominent. The largest cardiac oscillations at any level of the oesophagus were 6.5 (6.0) cmH_2_O in sitting compared to 18.4 (11.3) cmH_2_O in a supine position. The mean difference between the largest cardiac oscillations in sitting and supine positions was 11.9 cmH_2_O (95% CI 6.7–17.1 cmH_2_O) (Additional file 1: Figure S13). Average tidal variations in oesophageal pressure decreased in the part of oesophagus between 30 and 42 cm from the nostril but were otherwise not significantly affected by a change to the supine position.

## Discussion

This study shows a substantial variation within individual patients in both end-expiratory and tidal variation in oesophageal pressure and how changes in ventilator settings have different local effects on oesophageal pressure within the same patient. Both phenomena are difficult to recognize when a standard balloon catheter is used. Chest wall compressions unevenly affect oesophageal pressures, and supine position affects oesophageal pressures in a more complex way than just increasing the absolute oesophageal pressures.

Our results provide a unique analysis of oesophageal pressures during mechanical ventilation and important insights in its complexity. The methods used in the study, oesophageal balloon catheters used in critical care research and HRM used in gastroenterology are established methods although within different medical fields. Still, there are some uncertainties in the measurements that need to be addressed. In spite of being the filling volume recommended by the manufacturer, the use of 4 ml of air in the oesophageal balloon has previously been shown not to be optimal in every patient [13]. The HRM catheter is subject to pressure drift [25–27] partly due to the thermal effect and partly dependent on the length of measurement and level of pressure on the sensor. In this study, it is a minor problem since we focused mainly on the pressure differences at a certain moment and local effects of changes in ventilator settings. In order to minimize the effects of pressure drift, a catheter previously used in more than 100 examinations was utilized as recommended, the measurements were kept shorter than 30–40 min and recommended thermal compensation was applied before analysis [26]. The lack of individual calibration of the volume in the balloon and the pressure drift of the HRM may affect the comparison between oesophageal pressures measured with HRM and the conventional balloon catheter (performed in 10 patients). Due to these factors, the comparison focuses on describing the differences in information obtained. What the actual differences in absolute end-expiratory pressure and tidal pressure variations between methods represent needs to be further explored in future studies. Measurements in spontaneously breathing patients were performed on outpatients suffering from symptoms from the gastric and oesophageal area. Little is known regarding the oesophageal pathology and its effect on oesophageal pressure measurements in respiratory research. Since the main focus was a comparison between sitting and supine positions, this is a minor issue. Also, any disturbances from the oesophageal pathology are more easily detected when HRM is used compared to the conventional oesophageal balloon catheters.

The large variation in absolute oesophageal pressure along the length of the oesophagus was described more than 60 years ago by Mead et al. [12]. The authors found a large variation in lung compliance when calculated from oesophageal pressures measured in different parts of the oesophagus with a short balloon (3 cm in length). The same variation was not seen when a long balloon (16 cm long in their study) was used. The length of the balloon on the Nutrivent™ catheter used in the present study is 10 cm. In 75% of the patients, the difference between the highest and lowest pressure in the oesophagus 30–42 cm from the nostril at baseline PEEP exceeded 10 cmH_2_O. When measuring oesophageal pressure, longer balloons are recommended [28] and as shown by Mead et al. [12] avoid the problem with varying oesophageal pressures. But due to its length, the balloon on the Nutrivent™ catheter is exposed to several different end-expiratory oesophageal pressures and ΔPES, which also change differently when ventilator settings are changed. The single pressure delivered from the oesophageal balloon is a mixture of many highly variable pressures, and the complexity of oesophageal pressures is partly obscured.

Measurements with the HRM catheter reveal several factors, which affect oesophageal pressures. The disappearance of cardiac oscillations during increased intrathoracic pressure (higher PEEP or at end-inspiration) may cause large changes in oesophageal pressure in some parts of the oesophagus, often opposite to the changes in other parts. These effects indicate that the cardiovascular system potentially has a profound influence locally on both end-expiratory and tidal variations in oesophageal pressure. When changing from sitting to supine position, cardiac oscillations increased as also described by Baydur et al. [29]. Since areas with large cardiac oscillations seem more susceptible to inverse changes in oesophageal pressure due to the changes in intrathoracic pressure, larger cardiac oscillations might indicate a more pronounced influence from the cardiovascular system in a supine position. The larger variability in end-expiratory oesophageal pressure in the supine position compared to sitting position supports the notion that oesophageal pressure in a supine position is a complex phenomenon affected by multiple factors. The previously described increase in end-expiratory oesophageal pressure in a supine position [15–18] was confirmed in our study (mean difference 12.3 cmH_2_O). Spontaneously breathing patients in a supine position generally had a positive oesophageal pressure even at end-inspiration as opposed to in a sitting position.

When confirming the correct placement of the balloon catheter, a positive pressure occlusion test [11, 30] is often used as well as the presence of visible cardiac oscillations [6, 10, 18]. Measurements with HRM catheter show that within a patient, the oesophageal pressures change unevenly during chest wall compressions. This could possibly be referred to the difference in pressure transmission from the thoracic wall to the oesophagus. The air-filled lungs distribute the increase in pressure evenly within the thoracic cage in contrast to the heart that directly compresses the oesophagus during chest wall compression. The difference in ΔPES due to chest wall compressions suggests that despite a ΔPES/ΔPAW ratio outside the recommended 0.8–1.2 [31], the oesophageal balloon could be in a correct position. Using cardiac oscillations as a marker of correct balloon placement may be problematic since even if they confirm that the balloon is located within the thorax, they indicate a position where oesophageal pressures to a high degree are influenced by the cardiovascular system.

## Conclusions

Oesophageal pressure is not dependent only on lung and chest wall mechanics but is affected by several other factors. The surprisingly large variation in pressure along the oesophagus, the increase in pressure in supine position and the unpredictable change in pressure as a response to increased PEEP or tidal inflation indicate a substantial influence on oesophageal pressures from the external factors such as the weight of the mediastinum and the cardiovascular system. The balloon on the conventional catheter is exposed to many very variable pressures along its length and does not reveal the described complexity. Oesophageal pressure measurements with conventional balloon catheters are an important clinical tool to guide ventilator settings in the ICU. The results presented in this study potentially have a significant impact on how oesophageal pressure measurements are used clinically to evaluate lung and chest wall mechanics.

Our findings also provide a possible explanation to why adjustment of PEEP according to absolute oesophageal pressure measured with a balloon catheter fails to improve patient outcome [32]. Since this is the first study of its kind, further measurements with HRM during mechanical ventilation and a more thorough comparison with the conventional balloon catheter are of paramount importance both to confirm the presented data and to improve the use of oesophageal pressures in the clinical situation.

## Additional file

Additional file 1: **Table S1.** M = male, F = female, ICU = intensive care unit, OR = operating room. TBI = traumatic brain injury, ICB = intracerebral haemorrhage, SAH = subarachnoid haemorrhage. PaO_2_ measured in mmHg. LH = lung-healthy. In patient 5, information about length is missing. Figures presented as *n* (%) or mean (SD). **Figure S1.** Oesophageal pressures measured with high-resolution manometry. Right panel: an illustration of the oesophagus with a red line representing the pressure measured 1 cm apart at a chosen time. Number and dots next to the oesophagus represent cm from the nostril. UES = upper oesophageal sphincter. LES = lower oesophageal sphincter. Left panel: pressures measured 2 cm apart along the oesophagus 24–46 cm from the nostril. Number on the left represents cm from the nostril, and bold numbers next to pressure curves is the pressure (in mmHg) measured at that level at a chosen time. Line representing the chosen time is not visible in the figure. **Figure S2.** Individual curves for each patient showing end-expiratory oesophageal pressure along the oesophagus at different PEEP levels. Measurements performed with the high-resolution manometry catheter. **Table S2.**Intra-individual variation in oesophageal pressure described with coefficient of variation, calculated as within-subject standard deviation divided by the mean (SD/mean). Values in the table represent the mean and median from all patients. Coefficient of variation is presented for end-expiratory pressures at different PEEP levels and for tidal variations in oesophageal pressure at different tidal volumes. 22 cm of oesophagus (ESO_TOT_) and oesophagus 30–42 cm from the nostril (ESO_LOW_) are presented separately, see the “Methods” section. **Figure S3.** Figure shows how an increase of PEEP effects oesophageal pressure in two representative patients. When PEEP is increased, cardiac oscillations decrease which at some levels causes oesophageal pressure to decrease, black arrows. At other levels the oesophageal pressure increases, red arrows. **Figure S4.** Change in end-expiratory oesophageal pressure after an increase of PEEP of ≈ 7 cmH_2_O. Oesophageal pressures measured with balloon catheter and HRM catheter respectively. Number in the centre of figure represents cm from the nostril. Measurements from representative patient. **Figure S5.** Change in end-expiratory oesophageal pressure and end-expiratory lung volume after an increase of PEEP of ≈ 7 cmH_2_O. End-expiratory airway pressure (PEEP), red curve, and end-expiratory oesophageal pressure, blue and black curve, increases and reaches the new level after the first breath when PEEP is changed on the ventilator. The end-expiratory lung volume, green, continues to increase for several breathes. The curve with oesophageal pressure measured with HRM (black curve) is from a chosen level (28 cm from the nostril) with a large increase in end-expiratory oesophageal pressure in contrast to the other levels in the patient, see additional Figure S4. **Figure S6.** Tidal changes in oesophageal pressure (ΔPES) along the length of the oesophagus during different sizes of tidal volumes. Measurements performed with the high resolution manometry catheter. Tidal inspiration starting from baseline PEEP level, see the “Results” section. **Figure S7.** Tidal changes in oesophageal pressure (ΔPES) along the length of the oesophagus at the same tidal volume (≈ 6 ml/kg IBW) but different PEEP. Measurements performed with the high-resolution manometry catheter. **Figure S8.**End-expiratory oesophageal pressure. Comparison between measurements from HRM catheter and balloon catheter. **Figure S9.** Tidal variation in oesophageal pressure (ΔPES). Comparison between measurements from HRM catheter and balloon catheter. Tidal inspiration starting from baseline PEEP level, see “Results” section. Red dotted line represents mean tidal change in oesophageal pressure calculated from pressures measured between 30 and 42 cm from the nostril with the high-resolution manometry catheter. **Table S3.** Difference between end-expiratory oesophageal pressure and between tidal variations in oesophageal pressure measured with HRM and conventional balloon catheter. In order to compare pressures in the same part of oesophagus, the values from HRM are a mean of the pressures 30–42 cm from the nostril which is the part of oesophagus where the 10 cm long balloon on the conventional catheter is placed. **Figure S10.** Comparison between end-expiratory oesophageal pressures measured with HRM and conventional balloon catheter in 10 patients according to Bland and Altman. Mean PEEP during measurements 10.4 cmH_2_O. Panel A) comparison between pressure from balloon catheter and HRM mean values 30–42 cm from the nostril. Mean bias 0.10 cmH_2_O (solid line), limits of agreement (± 2 SD) 9.14; − 8.94 cmH_2_O (dashed line). Panel B) comparison between pressure from balloon catheter and HRM at 40–41 cm from the nostril. Mean bias 3.30 cmH_2_O (solid line), limits of agreement (± 2 SD) 13.74; − 7.14 cmH_2_O (dashed line). **Figure S11.** Change in oesophageal pressure during a manual chest wall compression (used in positive pressure occlusion test to confirm correct placement of balloon catheter). Measurements performed with HRM catheter. **Figure S12.**End-expiratory oesophageal pressure in spontaneously breathing patient. Comparison between sitting and supine positions. Measurements performed with HRM catheter. **Figure S13.** Figure shows how a change from sitting to supine position affects oesophageal pressure in a representative patient. In supine position the oesophageal pressure is higher and the cardiac oscillations are larger. (PDF 873 kb)

## Data Availability

The datasets used and/or analysed during the current study are available from the corresponding author on reasonable request.
