# Supplementary material for: Detailed measurements of oesophageal pressure during mechanical ventilation with an advanced high-resolution manometry catheter
Source: Crit Care. 2019 Jun 13;23:217. doi: 10.1186/s13054-019-2484-8 (PMC6567527; doi:10.1186/s13054-019-2484-8)

Additional File 1

Detailed measurements of esophageal pressure during mechanical ventilation with an advanced high-resolution manometry catheter

Per Persson MD[1], Rebecca Ahlstrand MD PhD[2], Magni Gudmundsson MD[1], Alex de Leon MD PhD[1, 2], Stefan Lundin MD, PhD[1]
[1]Department of Anesthesiology and Intensive Care, Sahlgrenska University Hospital, Gothenburg, Sweden
[2] Department of Anesthesiology and Intensive Care, Örebro University Hospital, Örebro, Sweden

Content

Content		Page
Fig 1	Esophageal pressure measure with high resolution manometry	2
Table 1	Patient characteristics	3
Fig 2	End-expiratory esophageal pressure at different PEEP	4-7
Table 2	Coefficient of variation	8
Fig 3	Effect of increased PEEP on esophageal pressure	9
Fig 4	Change in end-expiratory esophageal pressure after a change of PEEP	10
Fig 5	Change in end-expiratory esophageal pressure and end-expiratory lung volume after a change of PEEP	11
Fig 6	Tidal change in esophageal pressure (ΔPES) during different tidal volumes	12-15
Fig 7	Tidal change in esophageal pressure (ΔPES) at different PEEP-levels	16-19
Fig 8	End-expiratory esophageal pressure: comparison between HRM catheter and balloon catheter	20-22
Fig 9	Tidal change in esophageal pressure: comparison between HRM catheter and balloon catheter	23-24
Table 3	Comparison of esophageal pressure measured with HRM and conventional balloon catheter	25
Fig 10	Comparison of end-expiratory esophageal pressure measured with HRM and conventional balloon catheter according to Bland and Altman	26
Fig 11	Positive occlusion test: Change in esophageal pressure during chest compression	27
Fig 12	End-expiratory esophageal pressure: comparison between sitting and supine position	28-29
Fig 13	Effect of body position of esophageal pressure	30

Additional Figure 1

Esophageal pressure measured with high resolution manometry

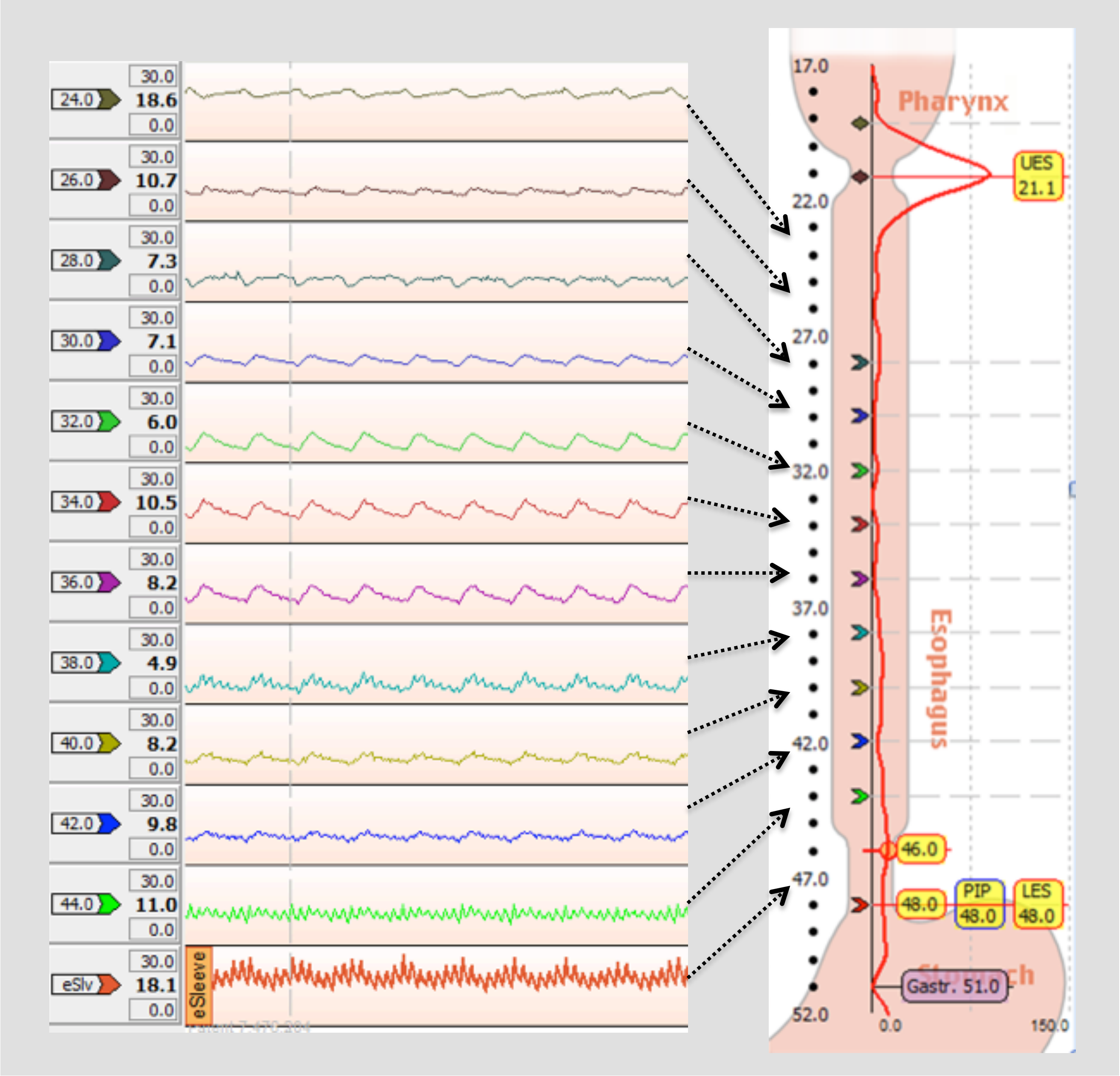

Supplementary Table 1
Patient Characteristics - mechanically ventilated patients

Patient	Sex (M / F)	Age (years)	Weight (kg)	BMI (kg/m²)	ICU or OR	Diagnosis / type of surgery	PaO2 / FiO2 ratio
1	M	59	75	19.7	ICU	TBI	>300
2	F	69	100	33.4	OR	Hysterectomy	LH
3	M	70	77	25.1	ICU	Pneumonia	>300
4	F	29	99	30.6	OR	Thyroidectomy	LH
5	M	48	91	-	ICU	TBI	>300
6	M	58	80	21.5	ICU	TBI	290
7	F	49	82	33.7	OR	Hysterectomy	LH
8	M	68	78	24.6	ICU	ICH	160
9	F	64	84	31.2	OR	Hand surgery	LH
10	F	27	67	23.7	OR	Cholecystectomy	LH
11	M	46	115	29.9	ICU	SAH	>300
12	M	66	102	28.9	ICU	Meningitis	290
13	F	54	64	24.4	ICU	Goodpasture syndrome	80
14	M	68	94	28.1	ICU	Cardiac arrest	200
15	M	54	84	27.1	ICU	SAH	200
16	F	82	56	22.7	ICU	ICH	270
17	F	56	92	35.9	ICU	SAH	160
18	M	70	86	26.8	ICU	ICH	190
19	M	24	53	17.3	ICU	ICH	>300
20	F	61	61	19.9	ICU	TBI	280
	Female 9/20 (45%)	56.1 (15.5)	82.0 (16.3)	26.6 (5.2)	ICU=15 (75%) OR=5 (25%)		250 (86)

M=Male, F=Female
ICU=Intensive Care Unit, OR= Operating Room
TBI=Traumatic Brain Injury, ICB=Intracerebral Haemorrhage, SAH=Subarachnoid Haemorrhage
PaO_2 measured in mmHg. LH= Lung Healthy. In patient 5 information about length is missing.
Figures presented as n(%) or mean(SD)

Supplementary Figure 2
End-expiratory esophageal pressure at different PEEP-levels

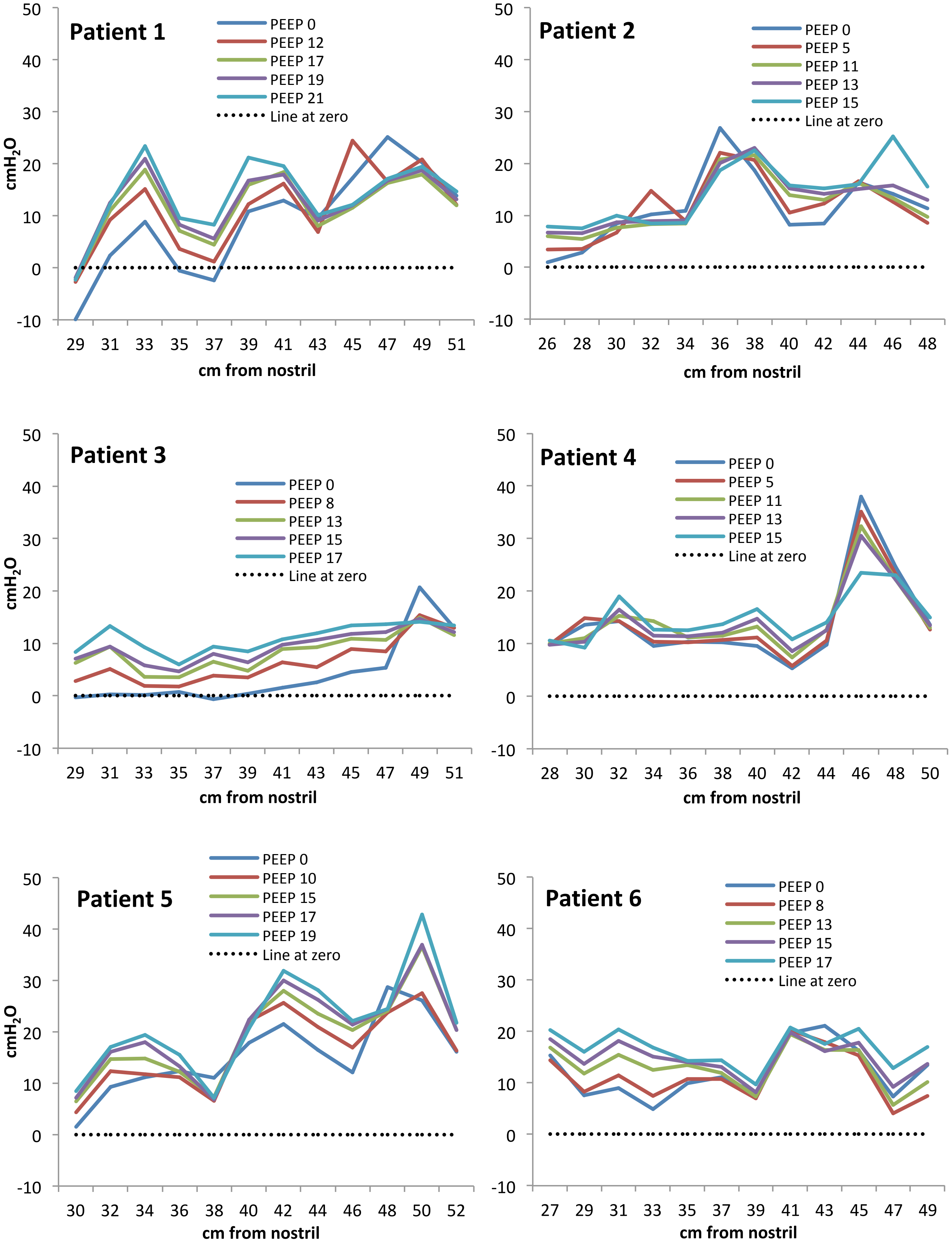

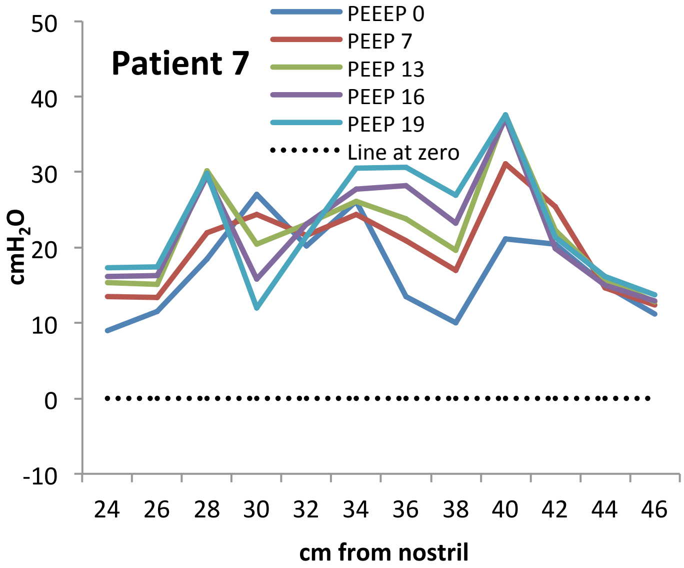
Patient 7
PEEEP 0
PEEP 7
PEEP 13
PEEP 16
PEEP 19
Line at zero
cmH2O
50
40
30
20
10
0
-10
24 26 28 30 32 34 36 38 40 42 44 46
cm from nostril

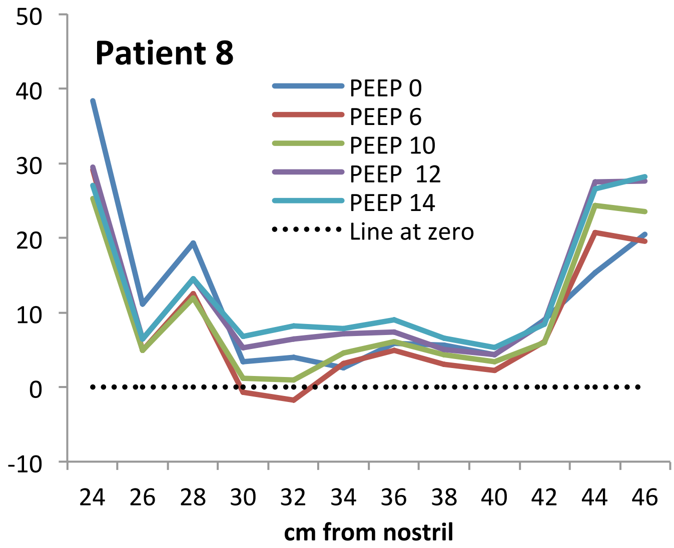
Patient 8
PEEP 0
PEEP 6
PEEP 10
PEEP 12
PEEP 14
Line at zero
50
40
30
20
10
0
-10
24 26 28 30 32 34 36 38 40 42 44 46
cm from nostril

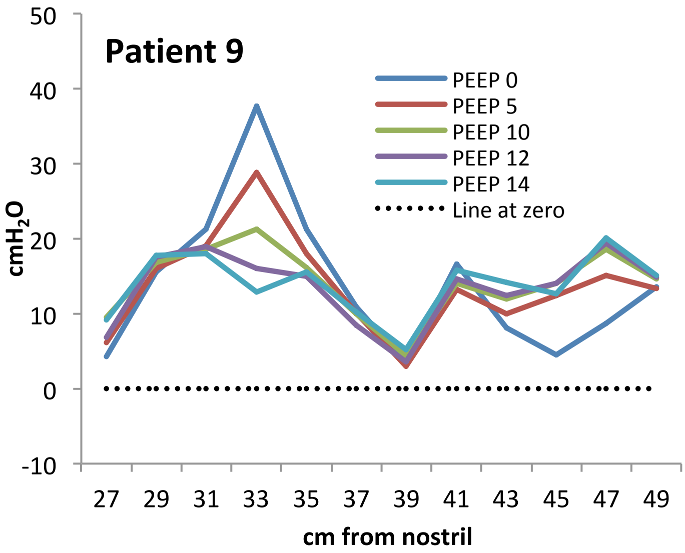
Patient 9
PEEP 0
PEEP 5
PEEP 10
PEEP 12
PEEP 14
Line at zero
cmH2O
50
40
30
20
10
0
-10
27 29 31 33 35 37 39 41 43 45 47 49
cm from nostril

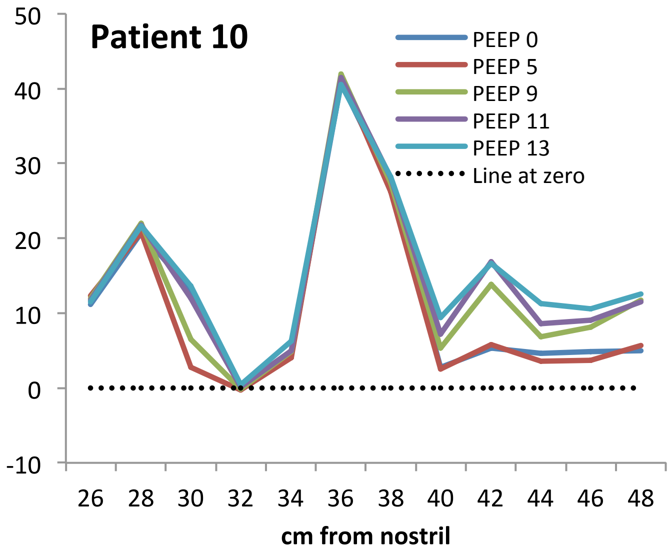
Patient 10
PEEP 0
PEEP 5
PEEP 9
PEEP 11
PEEP 13
Line at zero
50
40
30
20
10
0
-10
26 28 30 32 34 36 38 40 42 44 46 48
cm from nostril

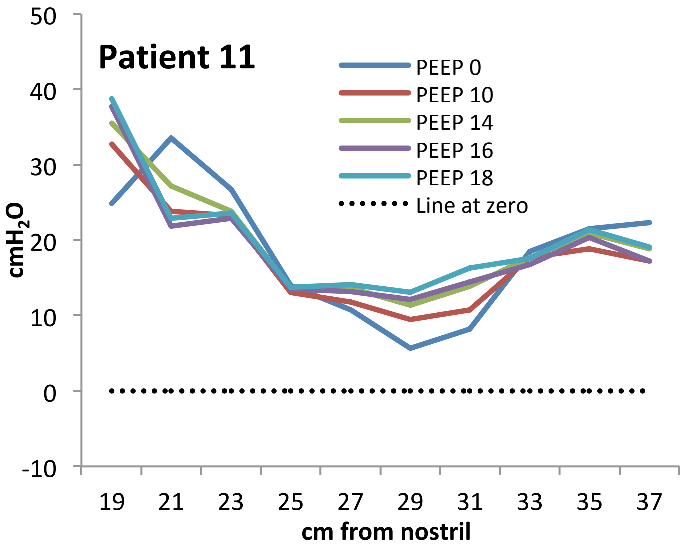
Patient 11
PEEP 0
PEEP 10
PEEP 14
PEEP 16
PEEP 18
Line at zero
cmH2O
50
40
30
20
10
0
-10
19 21 23 25 27 29 31 33 35 37
cm from nostril

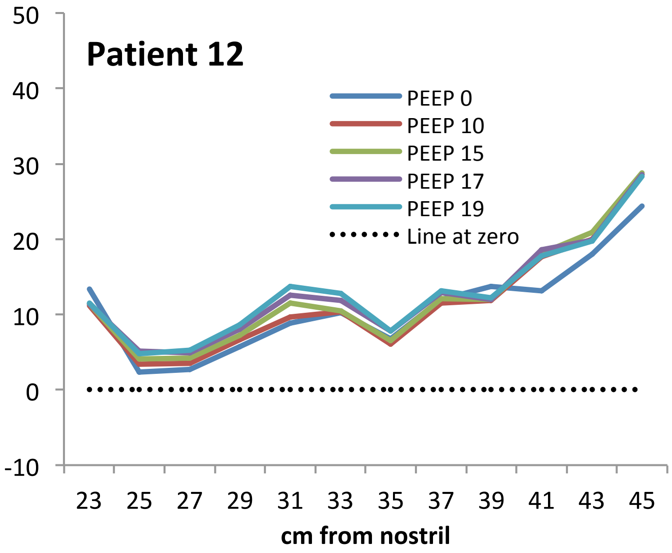
Patient 12
PEEP 0
PEEP 10
PEEP 15
PEEP 17
PEEP 19
Line at zero
50
40
30
20
10
0
-10
23 25 27 29 31 33 35 37 39 41 43 45
cm from nostril

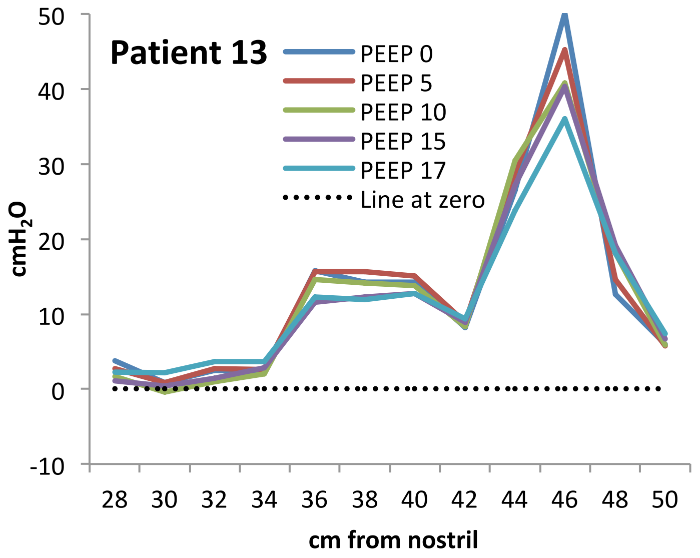
Patient 13
PEEP 0
PEEP 5
PEEP 10
PEEP 15
PEEP 17
Line at zero
cmH2O
cm from nostril

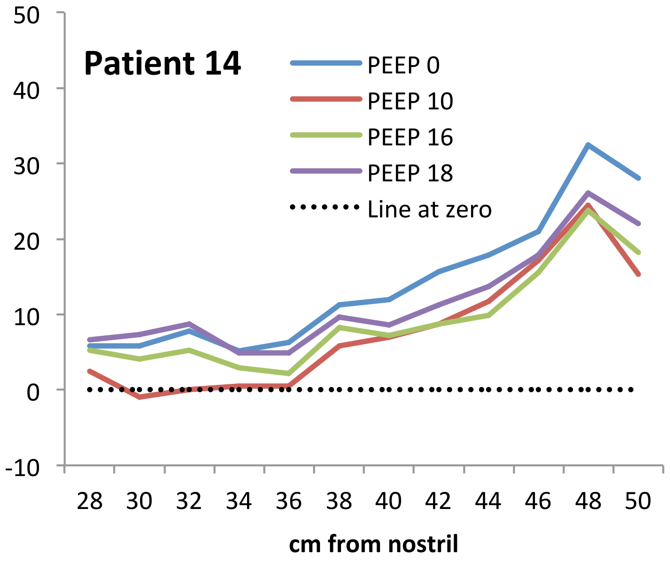
Patient 14
PEEP 0
PEEP 10
PEEP 16
PEEP 18
Line at zero
cm from nostril

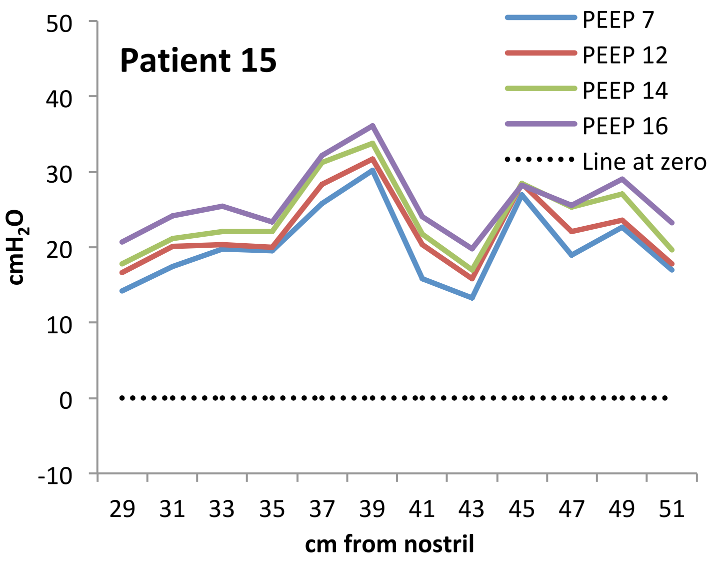
Patient 15
PEEP 7
PEEP 12
PEEP 14
PEEP 16
Line at zero
cmH2O
cm from nostril

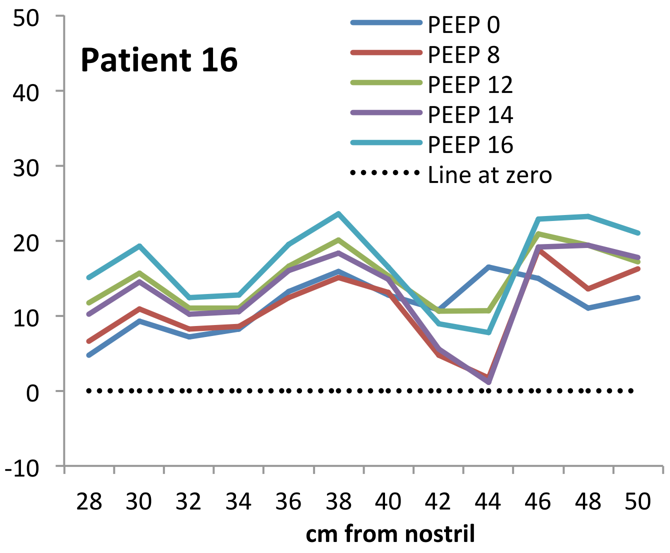
Patient 16
PEEP 0
PEEP 8
PEEP 12
PEEP 14
PEEP 16
Line at zero
cm from nostril

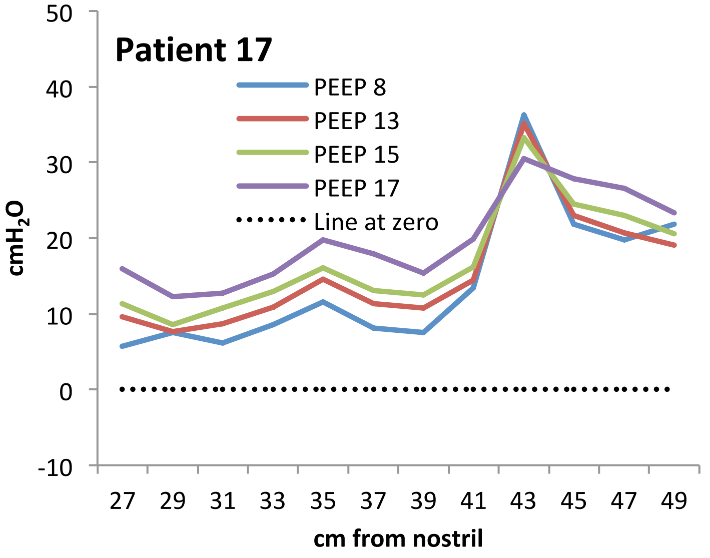
Patient 17
PEEP 8
PEEP 13
PEEP 15
PEEP 17
Line at zero
cmH2O
cm from nostril

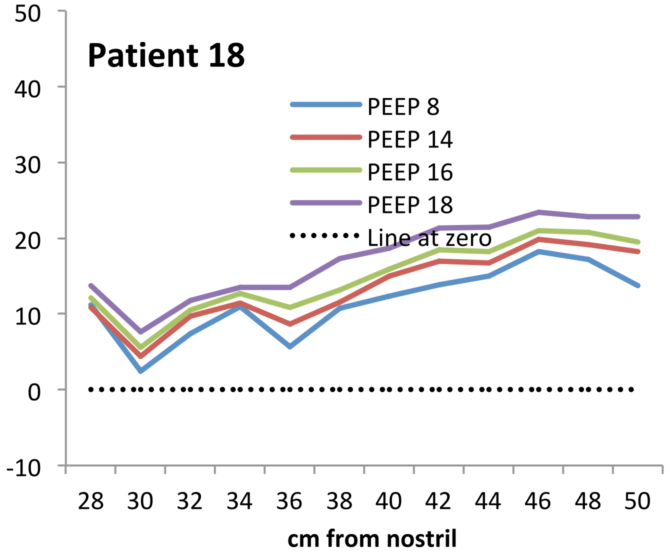
Patient 18
PEEP 8
PEEP 14
PEEP 16
PEEP 18
Line at zero
cm from nostril

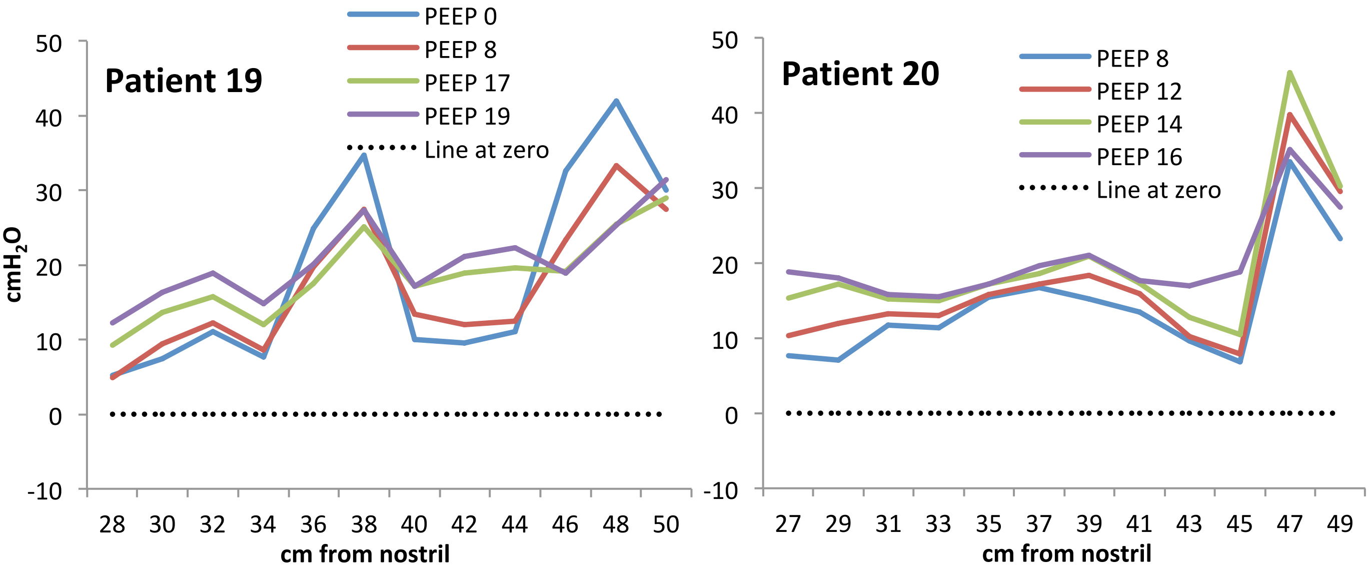
Patient 19
PEEP 0
PEEP 8
PEEP 17
PEEP 19
Line at zero
cmH2O
50
40
30
20
10
0
-10
28 30 32 34 36 38 40 42 44 46 48 50
cm from nostril
Patient 20
PEEP 8
PEEP 12
PEEP 14
PEEP 16
Line at zero
50
40
30
20
10
0
-10
27 29 31 33 35 37 39 41 43 45 47 49
cm from nostril

Supplementary Table 2.
Coefficient of variation

**End-expiratory esophageal pressure**	**Coefficient of variation**	
**Part of esophagus included**	**Low PEEP**<br>(n=20)<br>≈9 cmH_2O<br>Mean (SD)<br>Median (Min; max)	**High PEEP**<br>(n=20)<br>≈17 cmH_2O<br>Mean (SD)<br>Median (Min; max)
**22 cm of esophagus (ESO_{TOT})**	58 (24)%<br>50 (26; 104)%	41 (18)%<br>34 (18-84)%
**30-42 cm from nostril (ESO_{LOW})**	48 (29)%<br>44 (15; 127)%	32 (17)%<br>28 (12; 84)%
**Tidal change in esophageal pressure**	**Coefficient of variation**	
**Part of esophagus included**	**VT 1**<br>(n=20)<br>≈6 ml/kg<br>Mean (SD)<br>Median (Min; max)	**VT 3**<br>(n=19)<br>≈12 ml/kg<br>Mean (SD)<br>Median (Min; max)
**22 cm of esophagus (ESO_{TOT})**	150 (190)%<br>80 (38; 885) %	107 (66)%<br>93 (29; 214)%
**30-42 cm from nostril (ESO_{LOW})**	83 (52)%<br>82 (16; 196)%	151 (127)%<br>109 (49; 487)%

Supplementary Figure 3.
Effect of increased PEEP on esophageal pressure

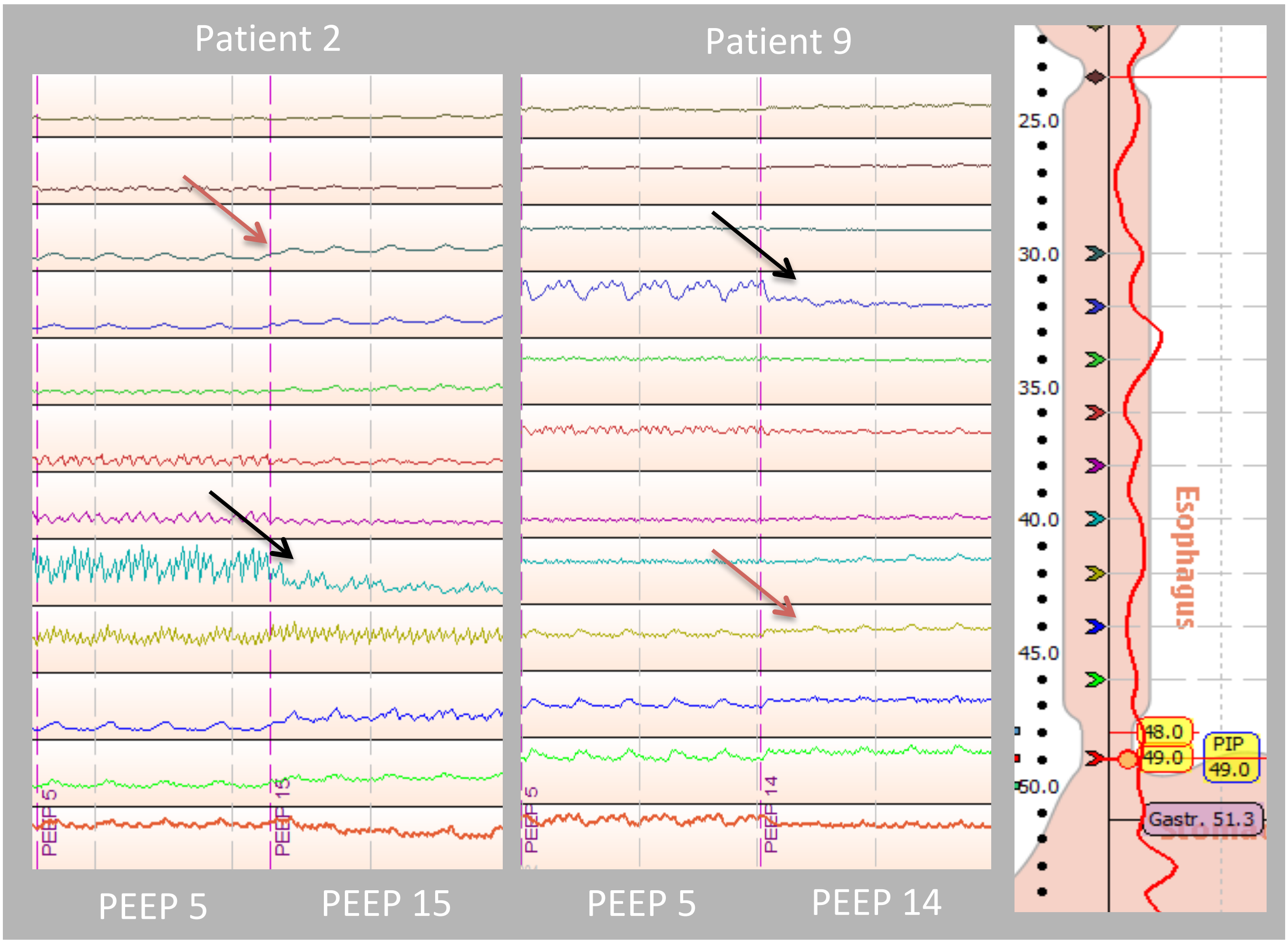

Supplementary Figure 4

Change in end-expiratory esophageal pressure after a change of PEEP

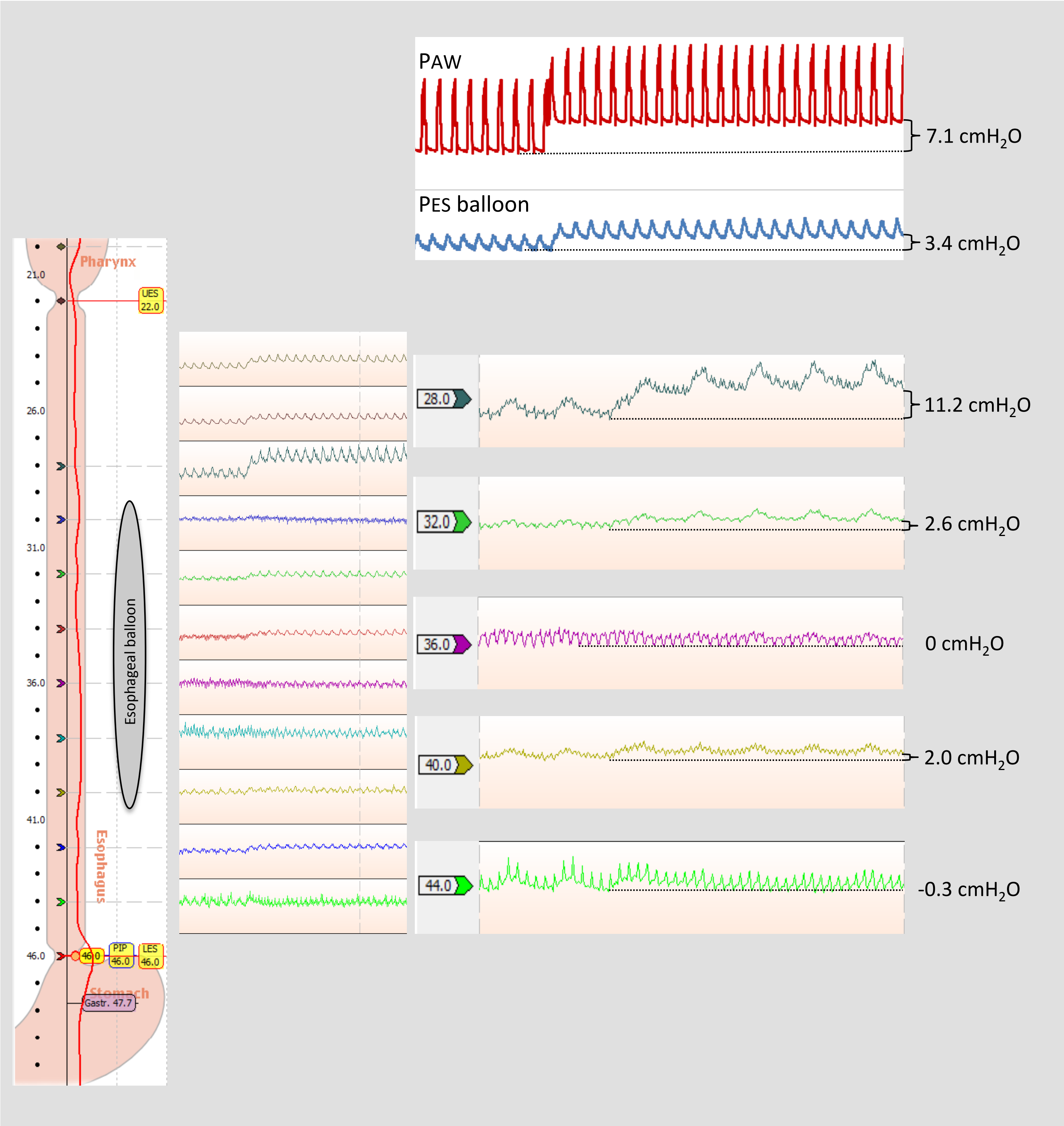

Supplementary Figure 5
Change in end-expiratory esophageal pressure and end-expiratory lung volume after a change of PEEP

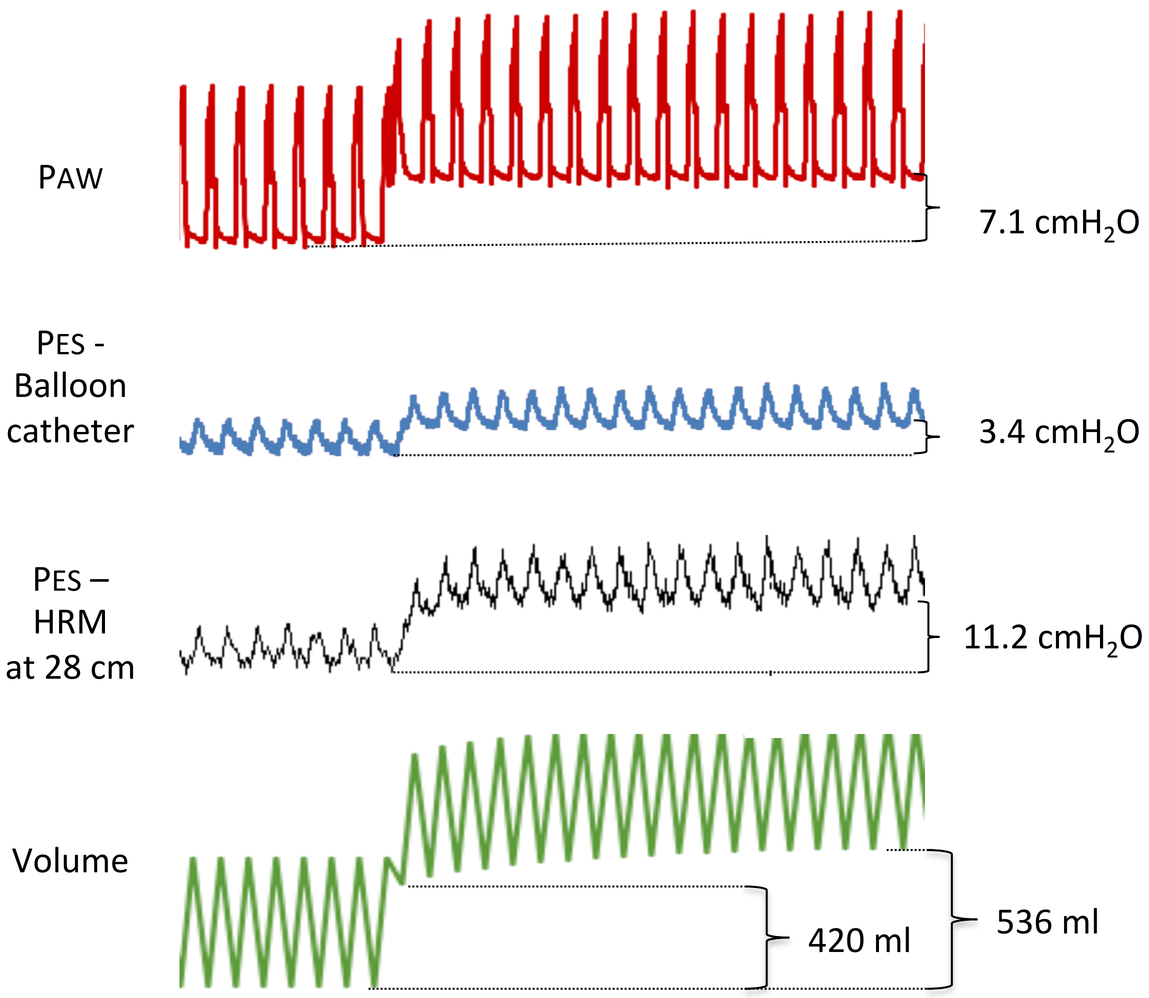

Supplementary Figure 6
Tidal change in esophageal pressure (ΔPES) during different tidal volumes

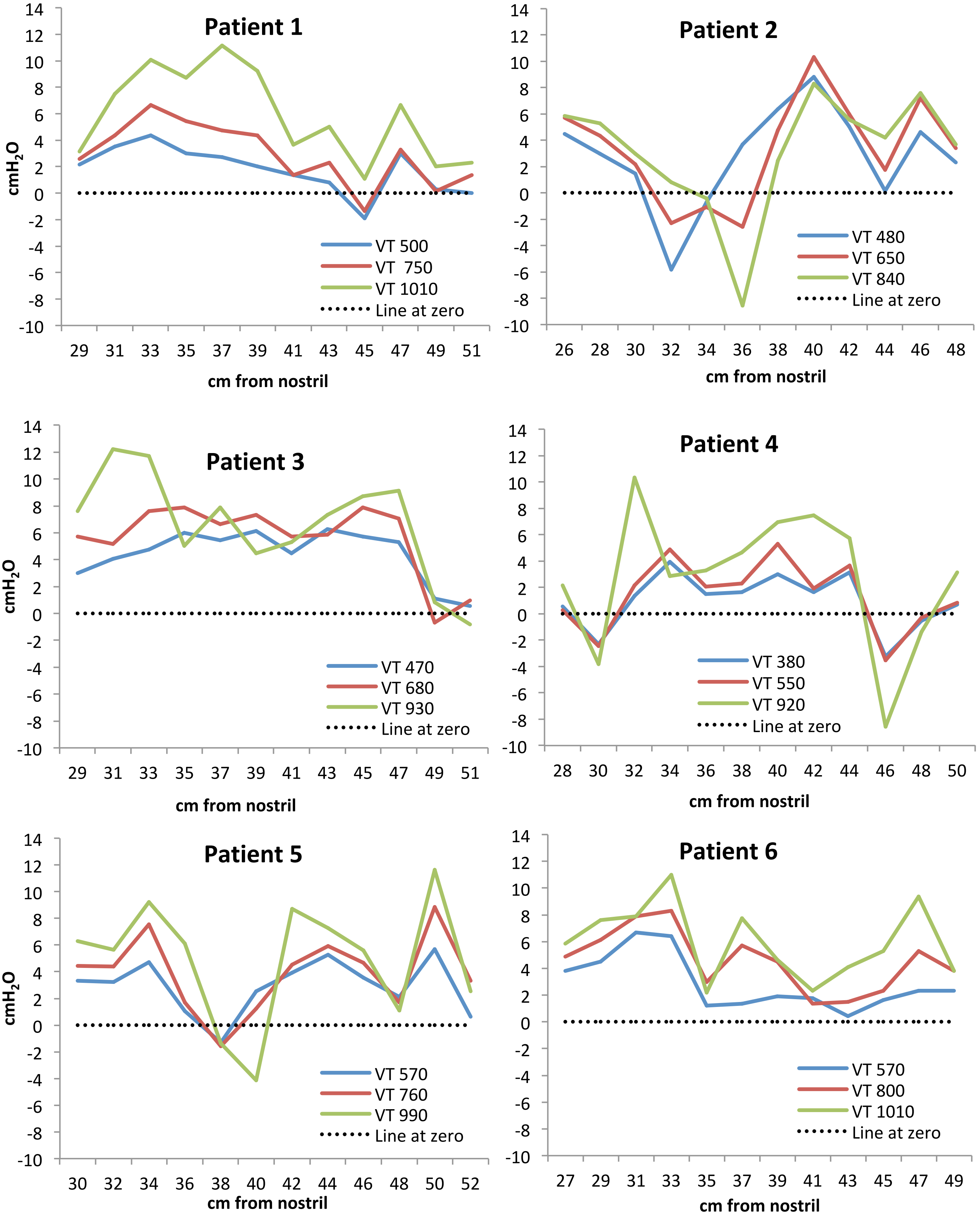

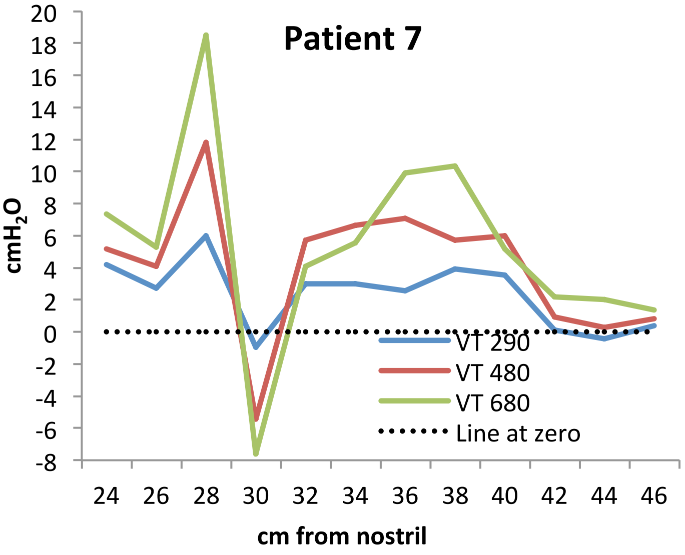
Patient 7
cmH_2O
cm from nostril
VT 290
VT 480
VT 680
Line at zero

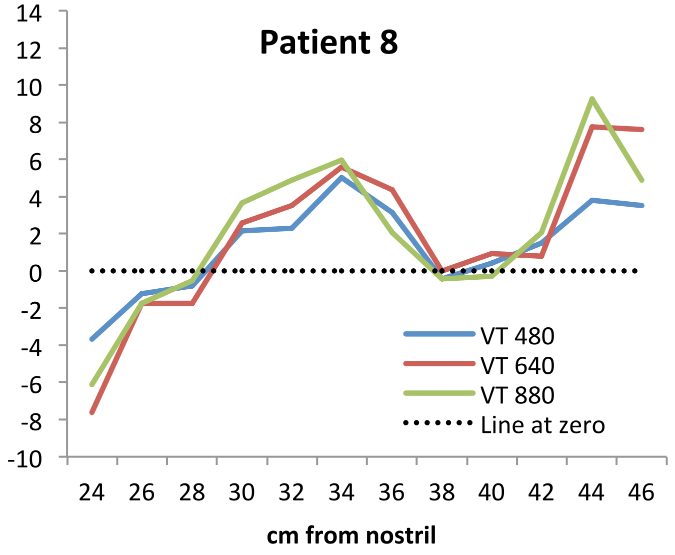
Patient 8
cm from nostril
VT 480
VT 640
VT 880
Line at zero

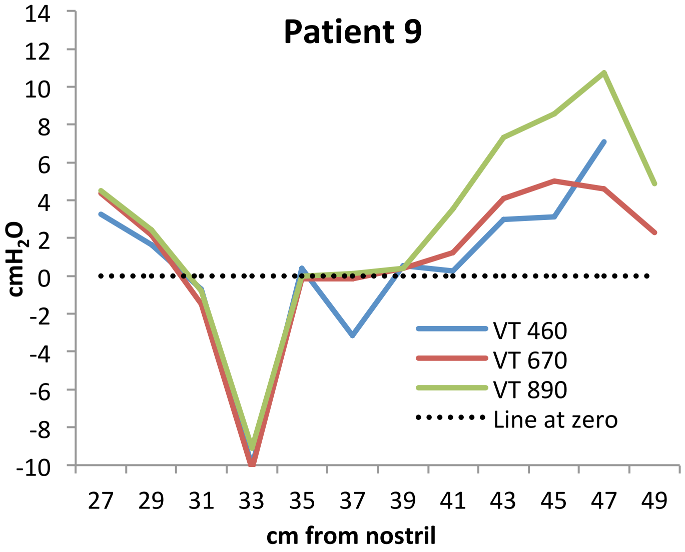
Patient 9
cmH_2O
cm from nostril
VT 460
VT 670
VT 890
Line at zero

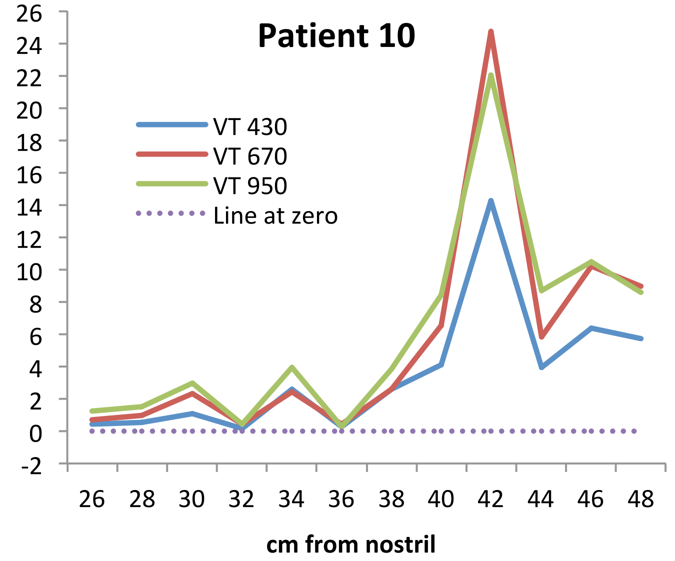
Patient 10
cm from nostril
VT 430
VT 670
VT 950
Line at zero

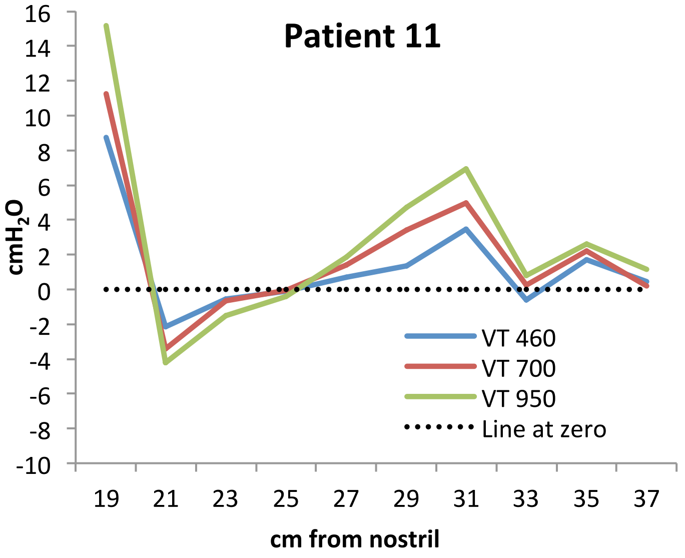
Patient 11
cmH_2O
cm from nostril
VT 460
VT 700
VT 950
Line at zero

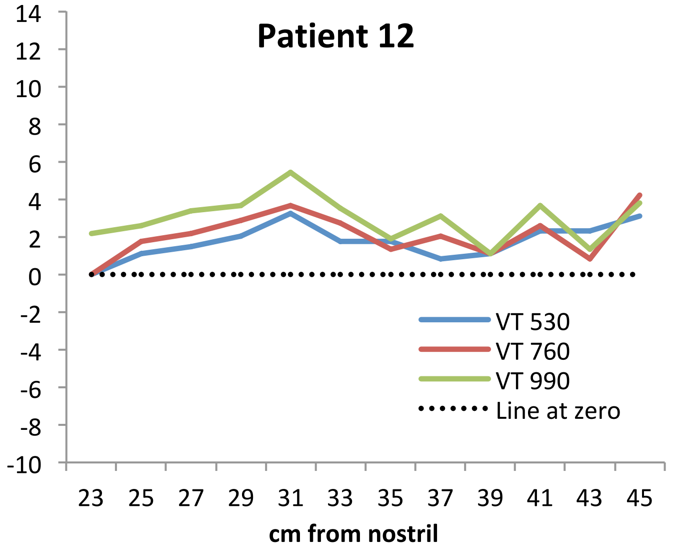
Patient 12
cm from nostril
VT 530
VT 760
VT 990
Line at zero

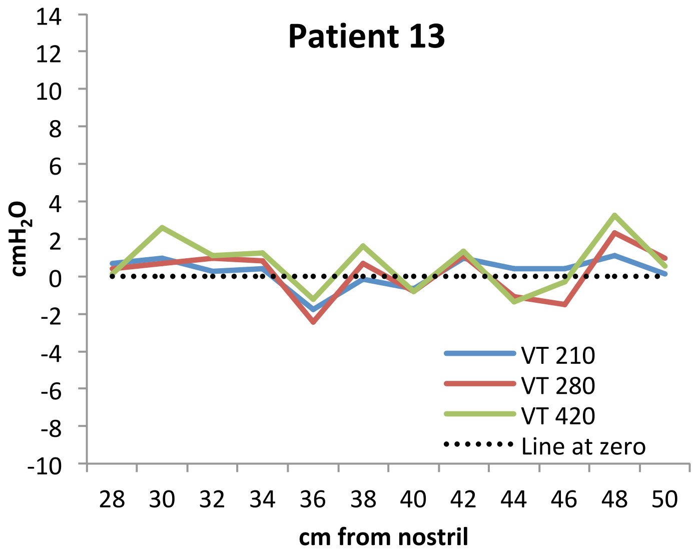
Patient 13
cmH2O
VT 210
VT 280
VT 420
Line at zero
cm from nostril

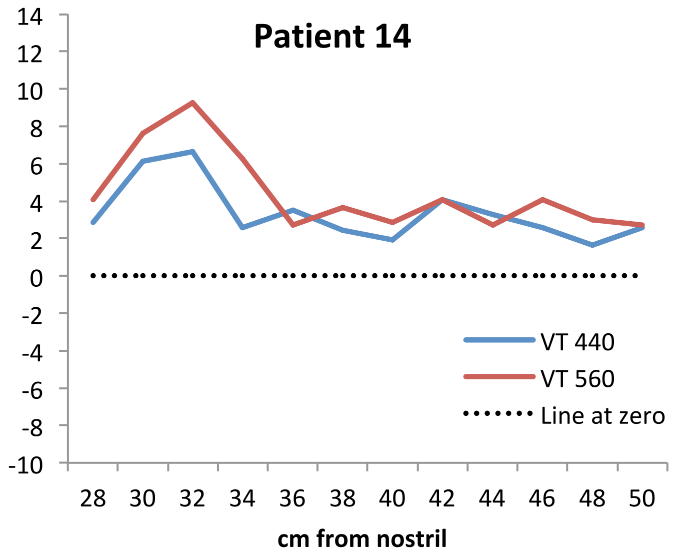
Patient 14
VT 440
VT 560
Line at zero
cm from nostril

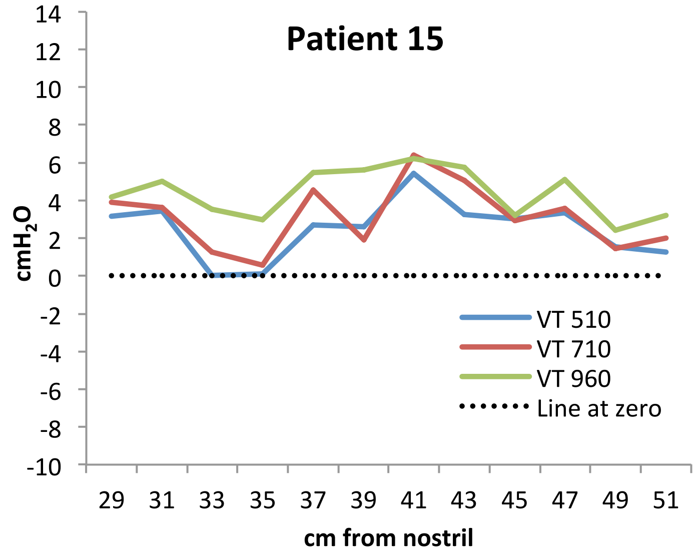
Patient 15
cmH2O
VT 510
VT 710
VT 960
Line at zero
cm from nostril

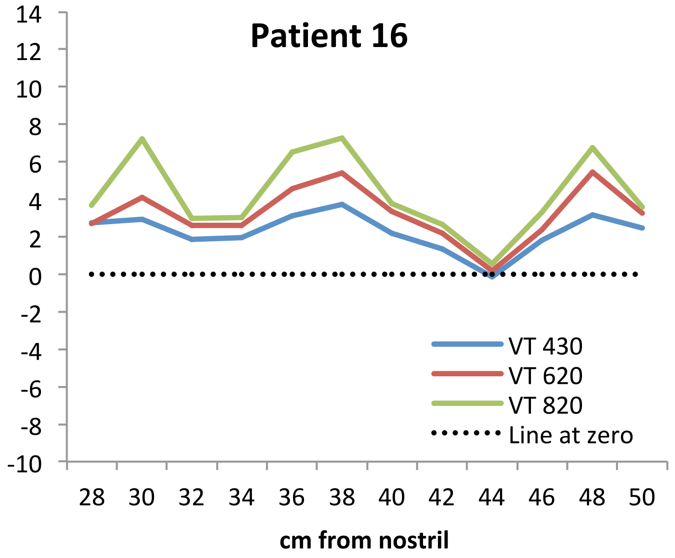
Patient 16
VT 430
VT 620
VT 820
Line at zero
cm from nostril

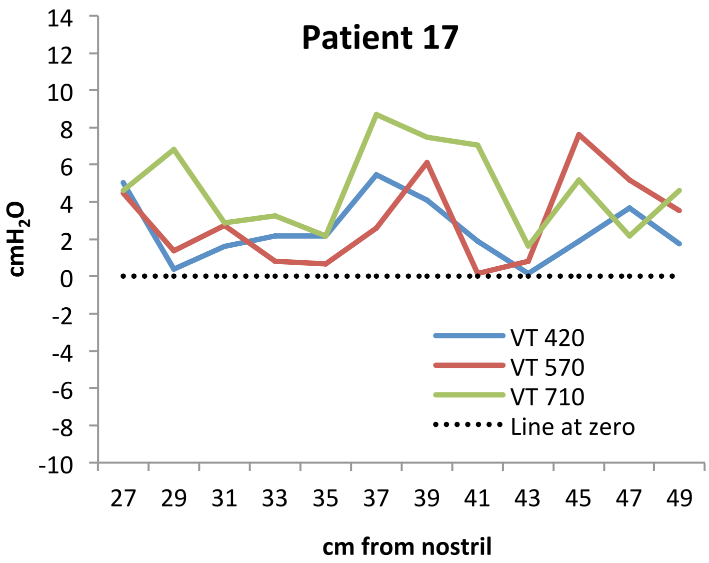
Patient 17
cmH2O
VT 420
VT 570
VT 710
Line at zero
cm from nostril

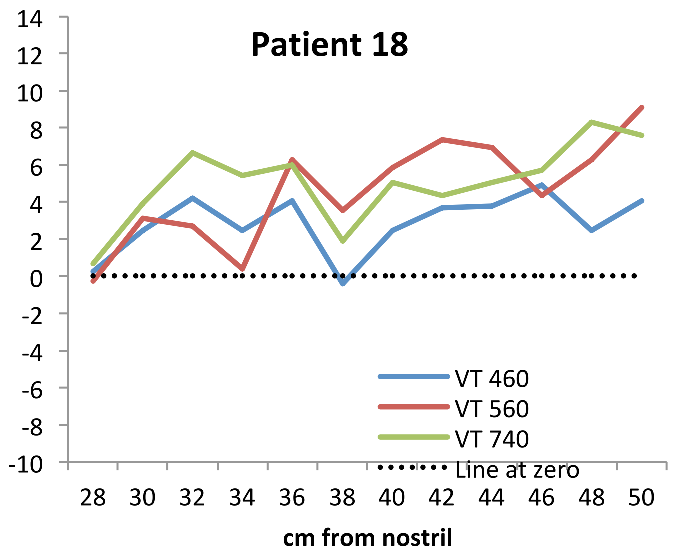
Patient 18
VT 460
VT 560
VT 740
Line at zero
cm from nostril

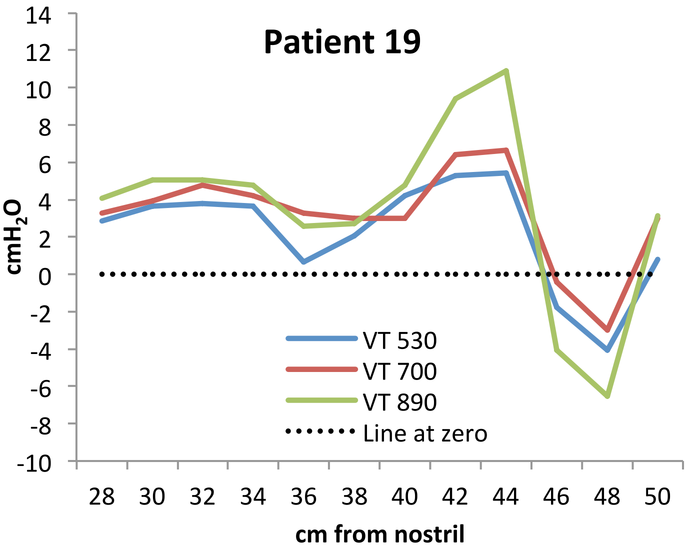
Patient 19
cmH2O
14
12
10
8
6
4
2
0
-2
-4
-6
-8
-10
28 30 32 34 36 38 40 42 44 46 48 50
cm from nostril
VT 530
VT 700
VT 890
Line at zero

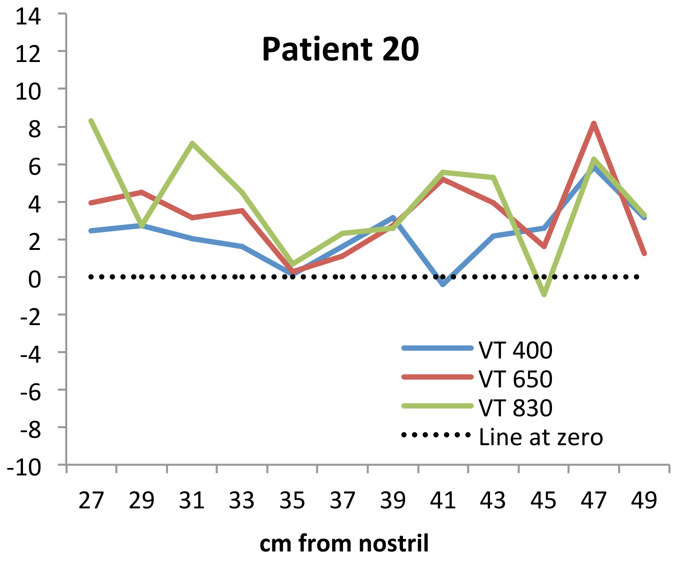
Patient 20
14
12
10
8
6
4
2
0
-2
-4
-6
-8
-10
27 29 31 33 35 37 39 41 43 45 47 49
cm from nostril
VT 400
VT 650
VT 830
Line at zero

Supplementary Figure 7

Tidal change in esophageal pressure (ΔPES) at different PEEP-levels

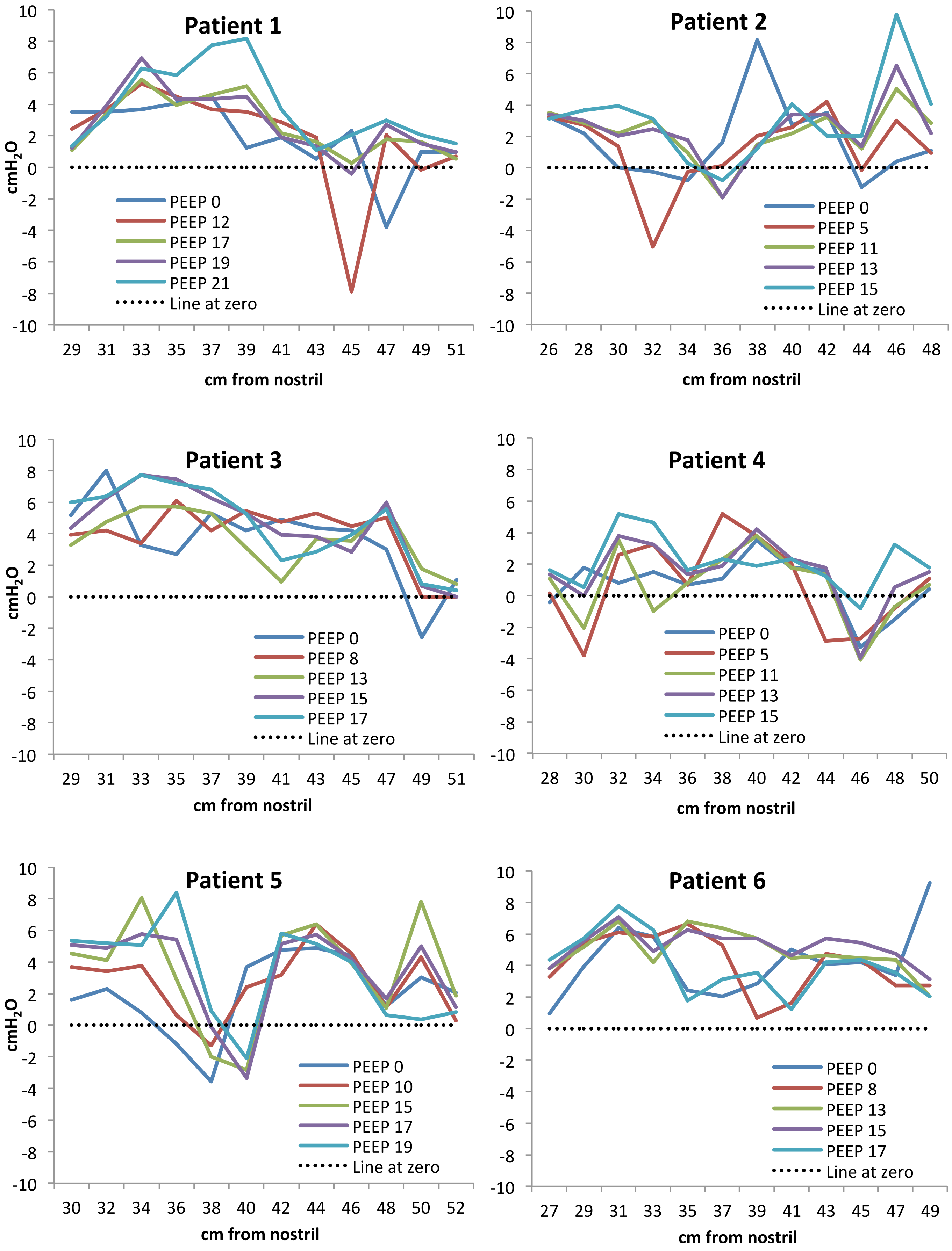

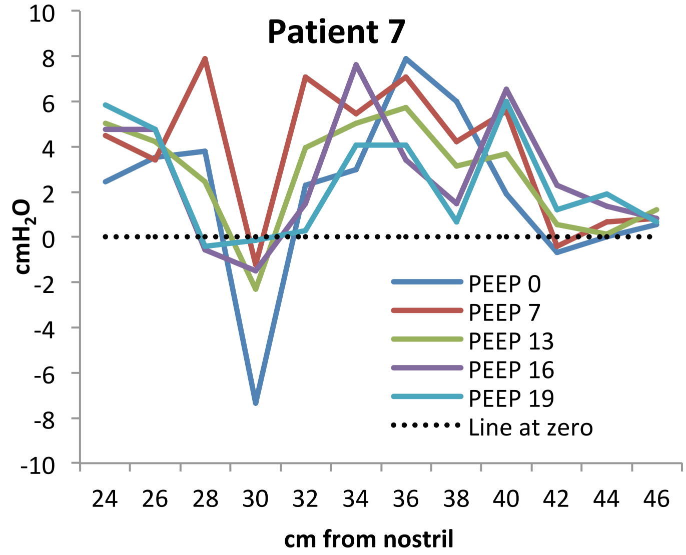
Patient 7
cmH2O
cm from nostril
PEEP 0
PEEP 7
PEEP 13
PEEP 16
PEEP 19
Line at zero

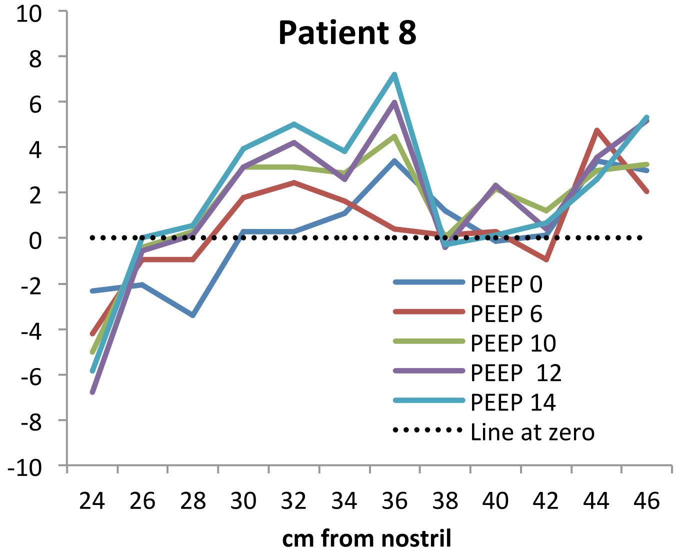
Patient 8
cm from nostril
PEEP 0
PEEP 6
PEEP 10
PEEP 12
PEEP 14
Line at zero

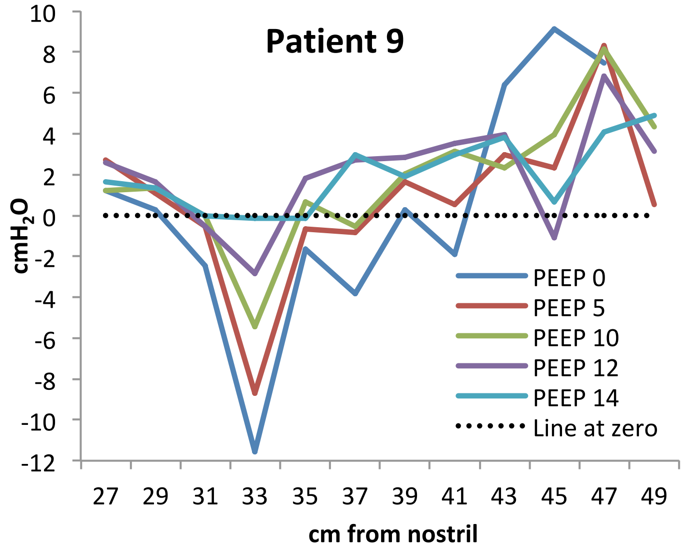
Patient 9
cmH2O
cm from nostril
PEEP 0
PEEP 5
PEEP 10
PEEP 12
PEEP 14
Line at zero

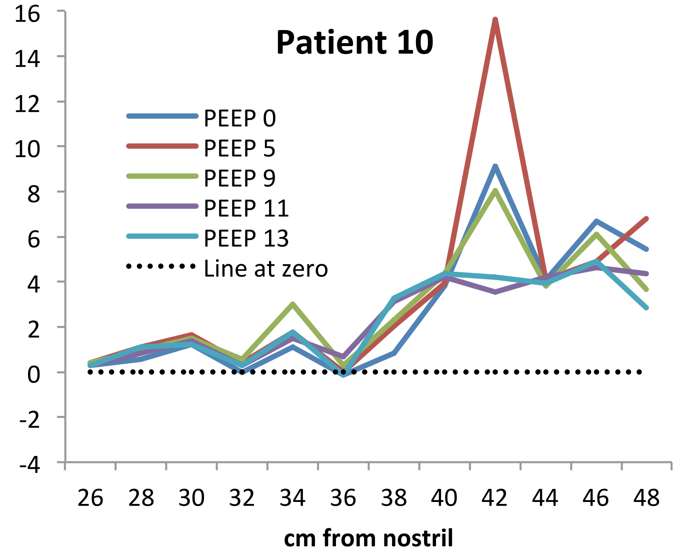
Patient 10
cm from nostril
PEEP 0
PEEP 5
PEEP 9
PEEP 11
PEEP 13
Line at zero

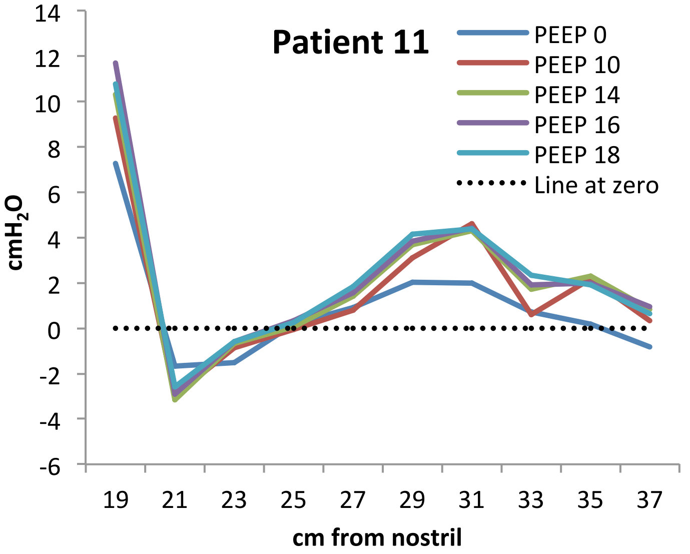
Patient 11
cmH2O
cm from nostril
PEEP 0
PEEP 10
PEEP 14
PEEP 16
PEEP 18
Line at zero

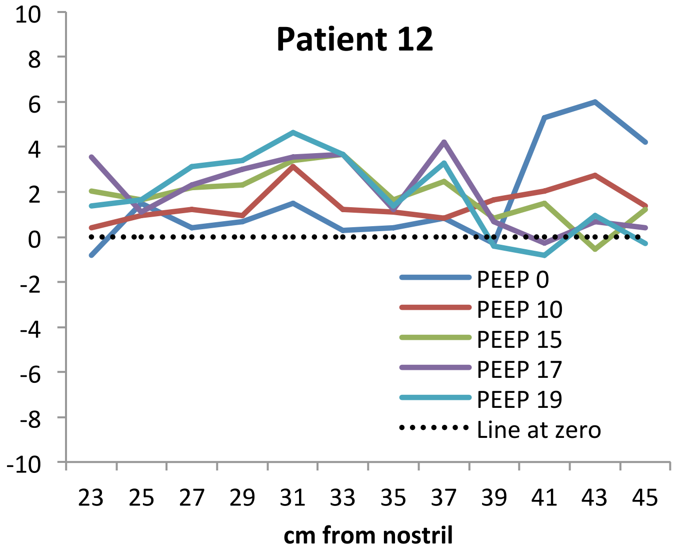
Patient 12
cm from nostril
PEEP 0
PEEP 10
PEEP 15
PEEP 17
PEEP 19
Line at zero

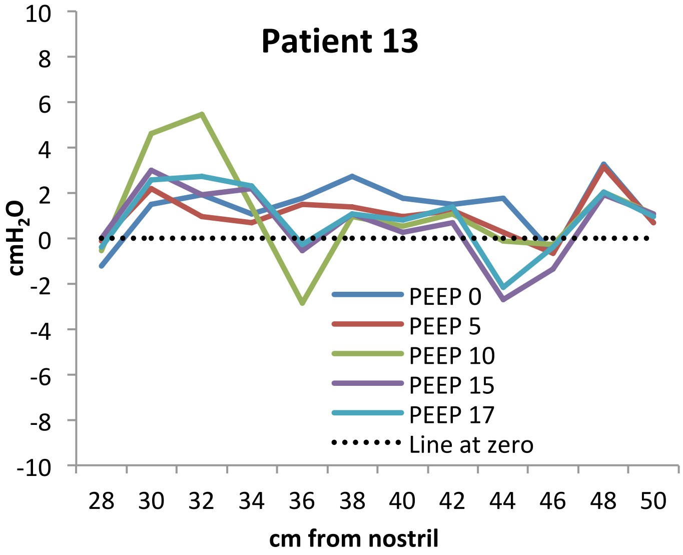
Patient 13
cmH2O
cm from nostril
PEEP 0
PEEP 5
PEEP 10
PEEP 15
PEEP 17
Line at zero

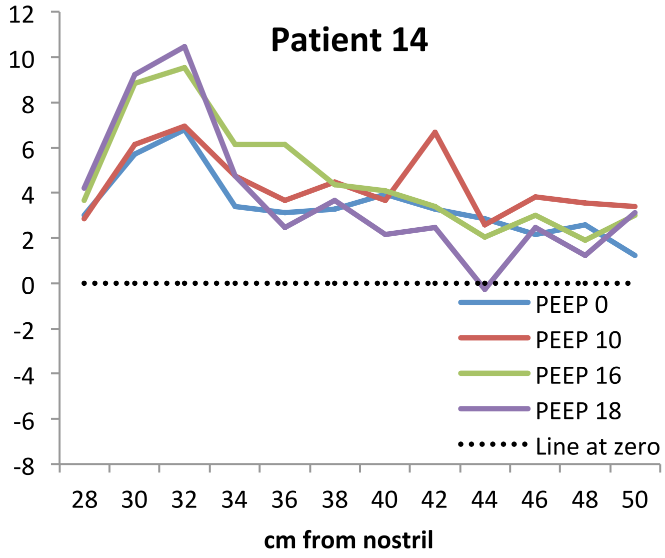
Patient 14
cm from nostril
PEEP 0
PEEP 10
PEEP 16
PEEP 18
Line at zero

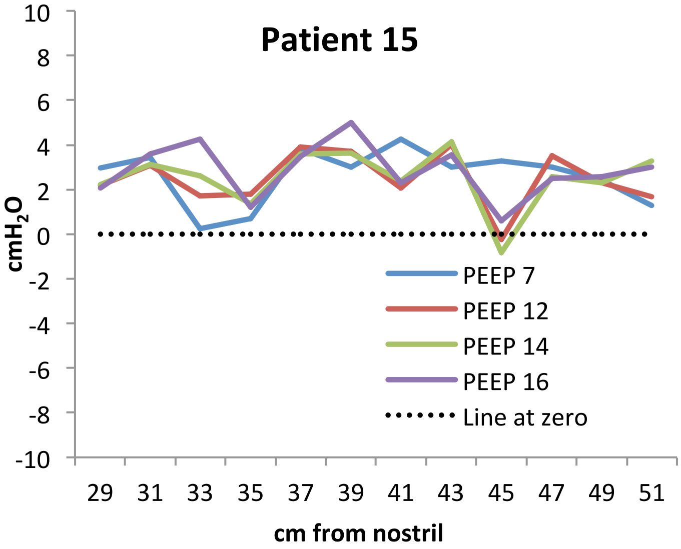
Patient 15
cmH2O
cm from nostril
PEEP 7
PEEP 12
PEEP 14
PEEP 16
Line at zero

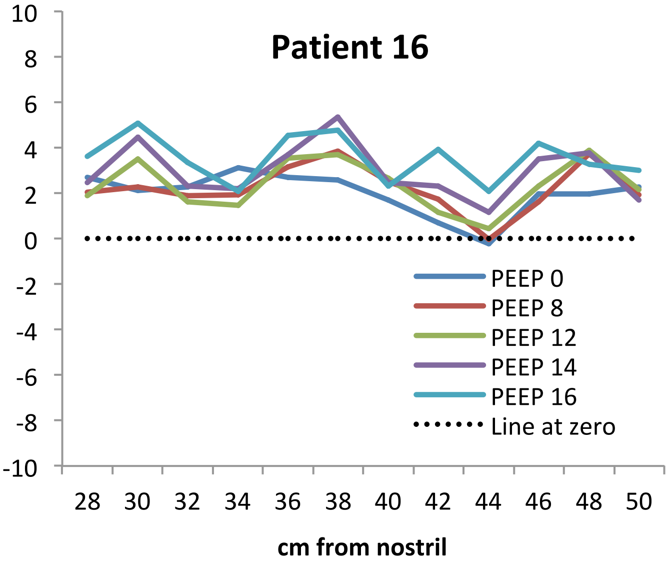
Patient 16
cm from nostril
PEEP 0
PEEP 8
PEEP 12
PEEP 14
PEEP 16
Line at zero

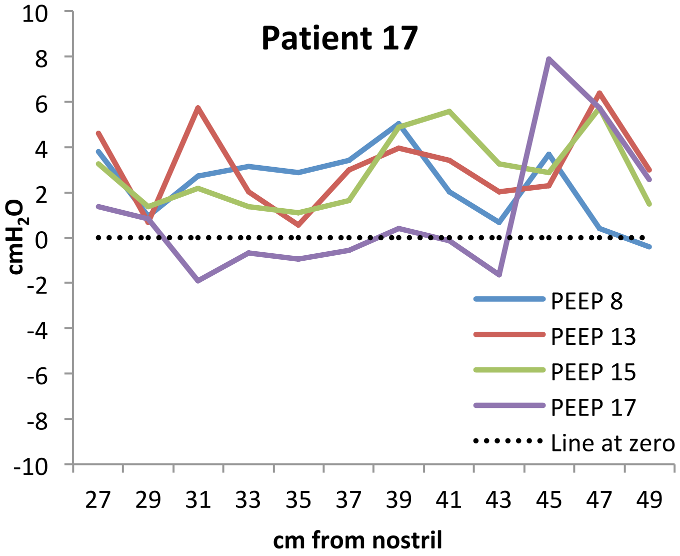
Patient 17
cmH2O
cm from nostril
PEEP 8
PEEP 13
PEEP 15
PEEP 17
Line at zero

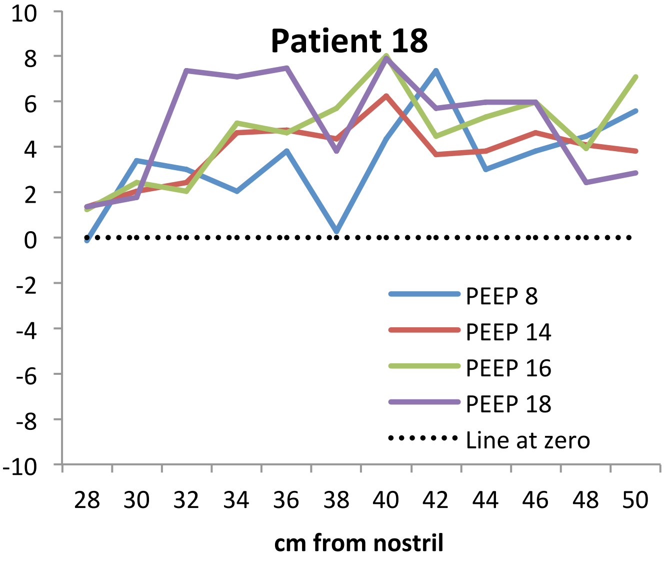
Patient 18
cm from nostril
PEEP 8
PEEP 14
PEEP 16
PEEP 18
Line at zero

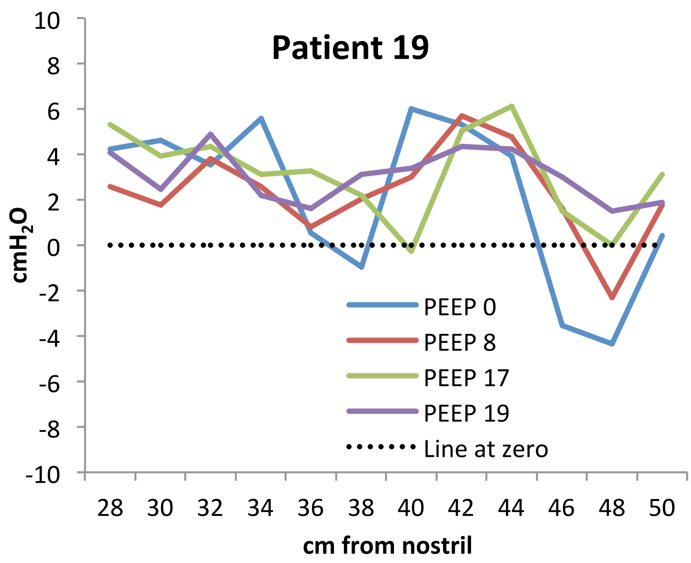
Patient 19
cmH2O
10
8
6
4
2
0
-2
-4
-6
-8
-10
PEEP 0
PEEP 8
PEEP 17
PEEP 19
Line at zero
28 30 32 34 36 38 40 42 44 46 48 50
cm from nostril

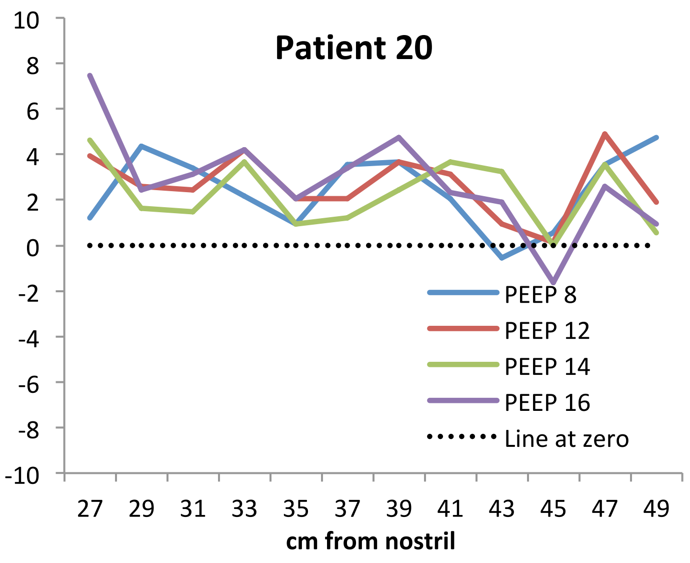
Patient 20
10
8
6
4
2
0
-2
-4
-6
-8
-10
PEEP 8
PEEP 12
PEEP 14
PEEP 16
Line at zero
27 29 31 33 35 37 39 41 43 45 47 49
cm from nostril

Supplementary Figure 8
End-expiratory esophageal pressure:
comparison between HRM catheter and balloon catheter

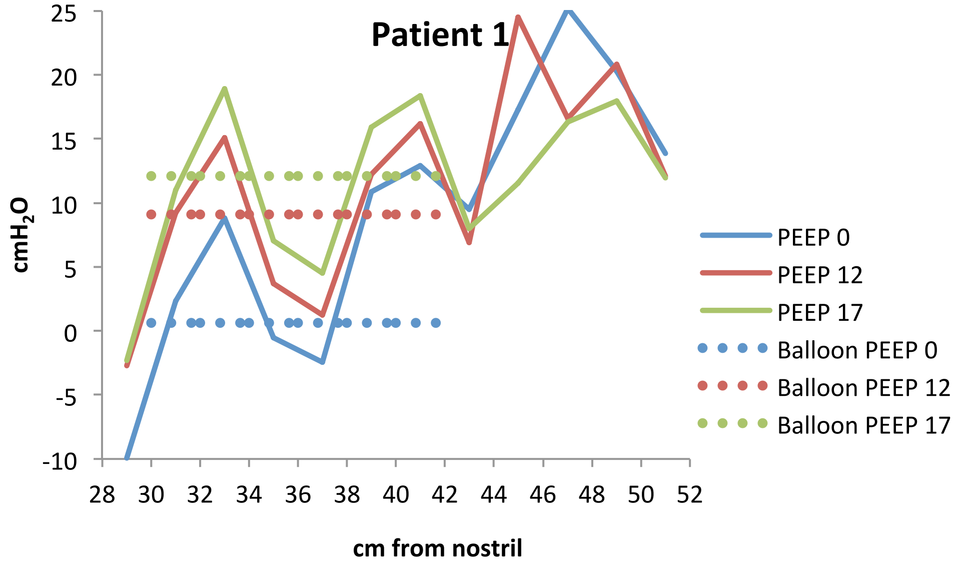

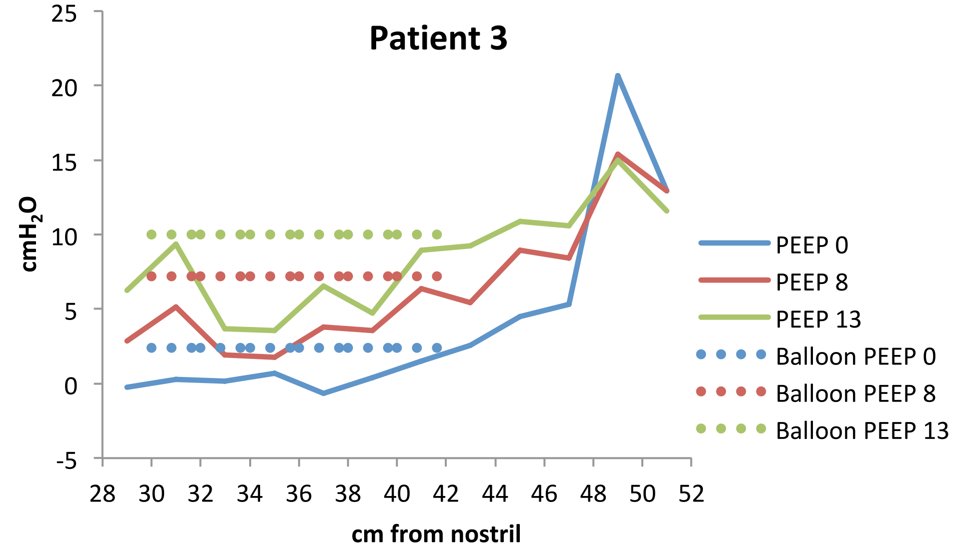

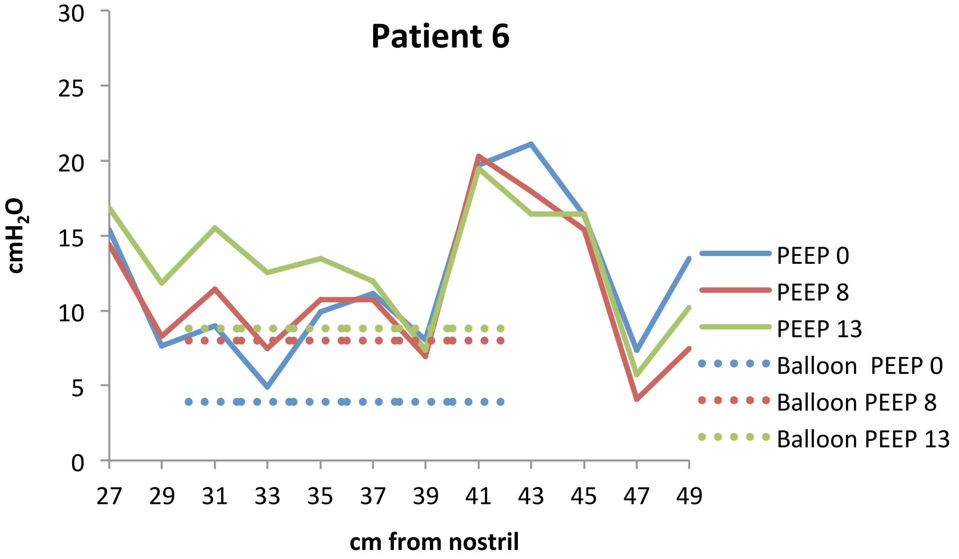

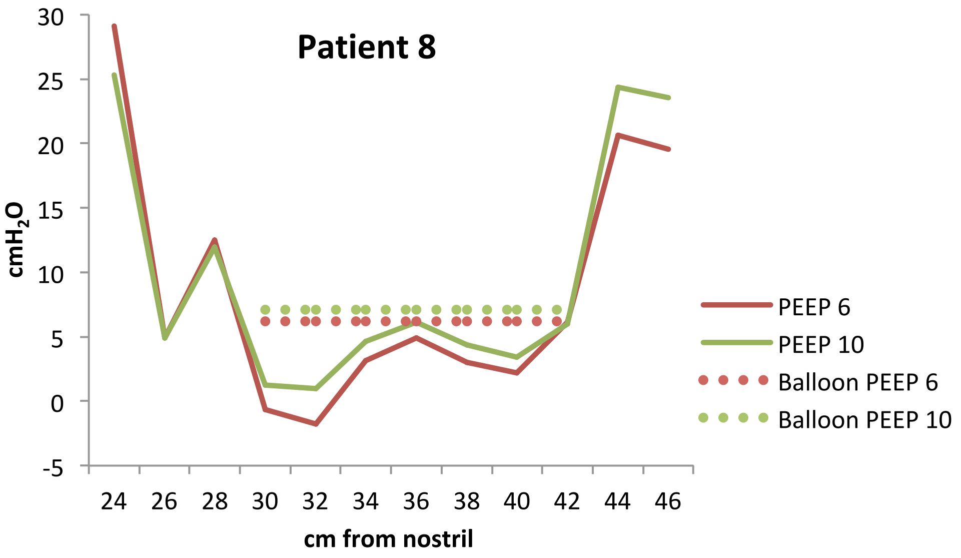

Patient 8
cmH2O
30
25
20
15
10
5
0
-5
24 26 28 30 32 34 36 38 40 42 44 46
cm from nostril
PEEP 6
PEEP 10
Balloon PEEP 6
Balloon PEEP 10

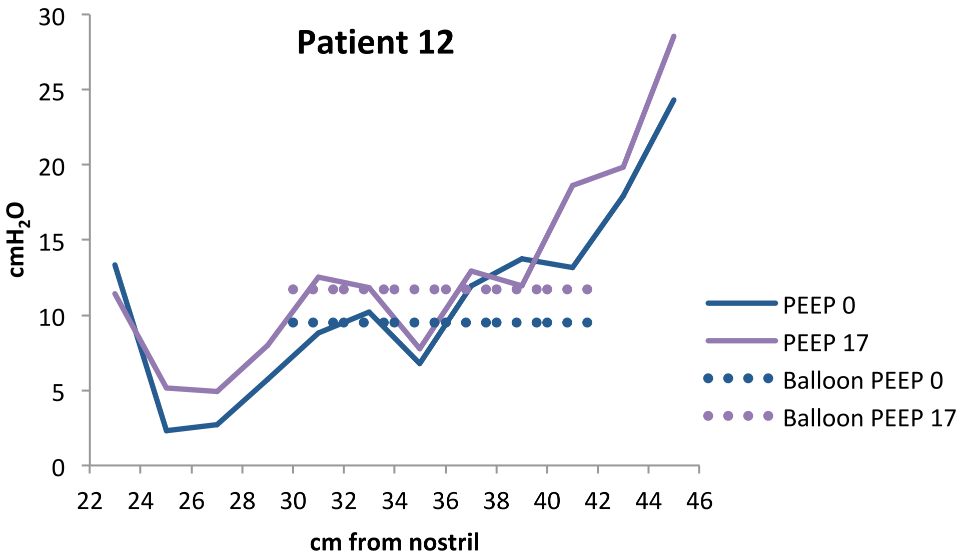

Patient 12
cmH2O
30
25
20
15
10
5
0
22 24 26 28 30 32 34 36 38 40 42 44 46
cm from nostril
PEEP 0
PEEP 17
Balloon PEEP 0
Balloon PEEP 17

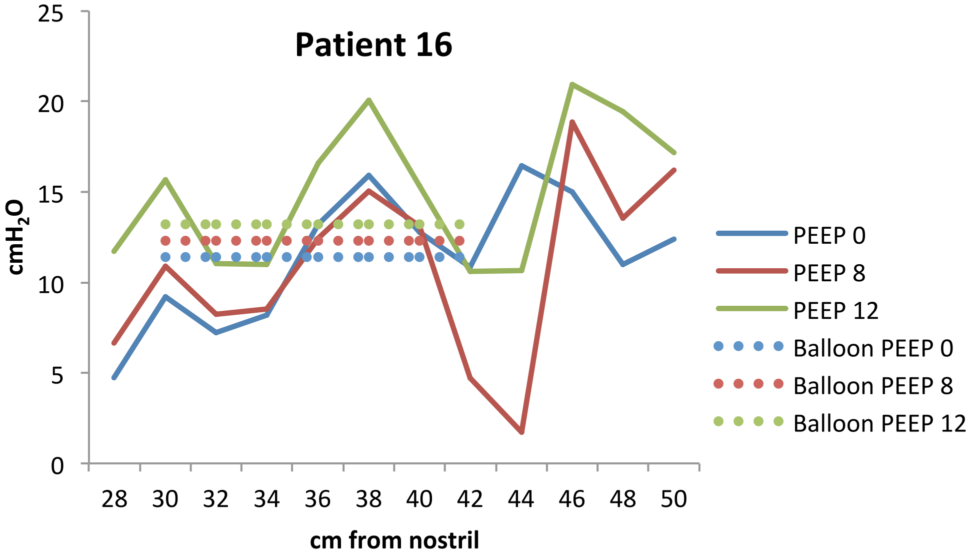

Patient 16
cmH2O
25
20
15
10
5
0
28 30 32 34 36 38 40 42 44 46 48 50
cm from nostril
PEEP 0
PEEP 8
PEEP 12
Balloon PEEP 0
Balloon PEEP 8
Balloon PEEP 12

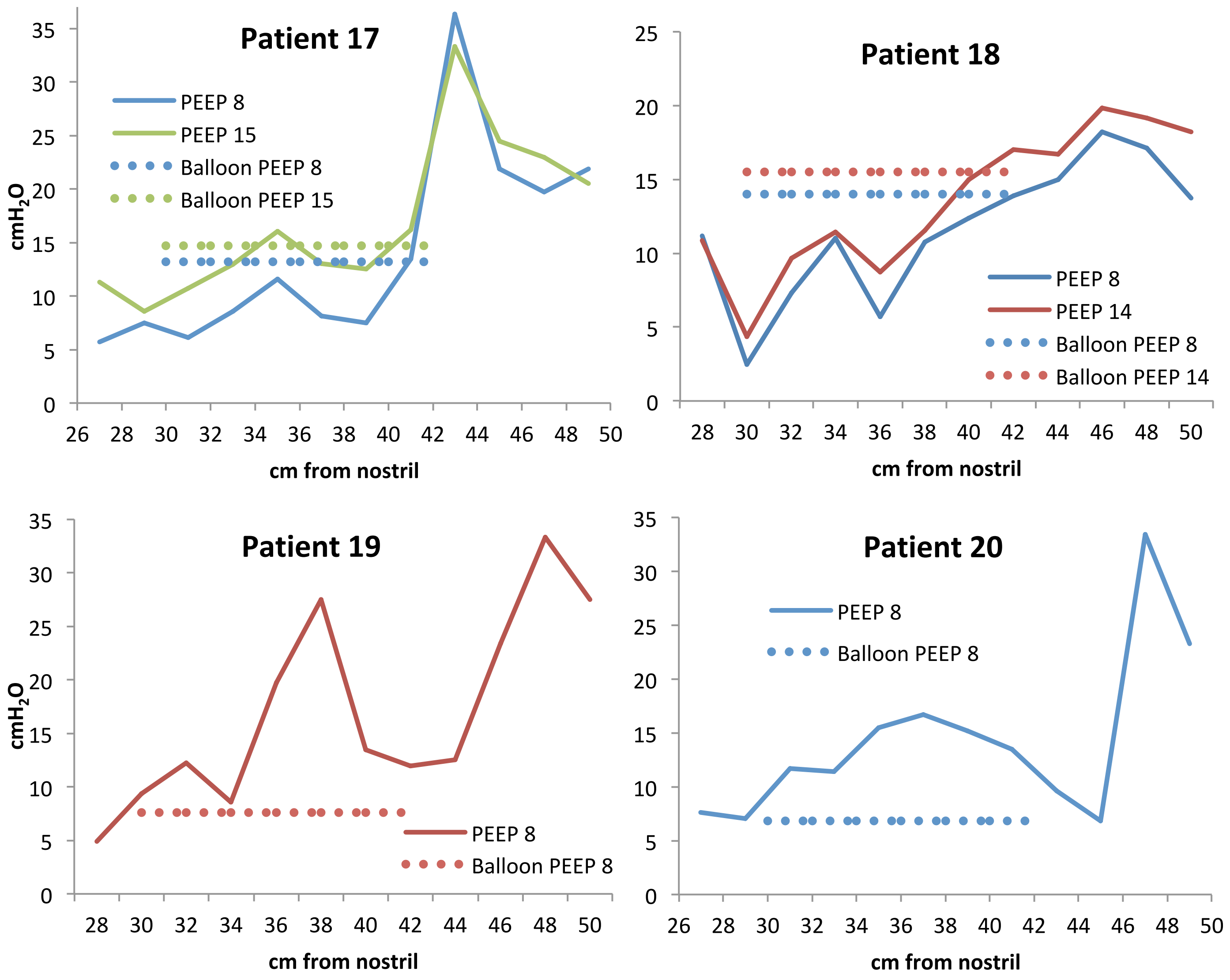
Patient 17
PEEP 8
PEEP 15
Balloon PEEP 8
Balloon PEEP 15
cmH2O
0 5 10 15 20 25 30 35
26 28 30 32 34 36 38 40 42 44 46 48 50
cm from nostril
Patient 18
PEEP 8
PEEP 14
Balloon PEEP 8
Balloon PEEP 14
0 5 10 15 20 25
28 30 32 34 36 38 40 42 44 46 48 50
cm from nostril
Patient 19
PEEP 8
Balloon PEEP 8
cmH2O
0 5 10 15 20 25 30 35
28 30 32 34 36 38 40 42 44 46 48 50
cm from nostril
Patient 20
PEEP 8
Balloon PEEP 8
0 5 10 15 20 25 30 35
26 28 30 32 34 36 38 40 42 44 46 48 50
cm from nostril

Supplementary Figure 9
Tidal change in esophageal pressure: comparison between HRM catheter and balloon catheter

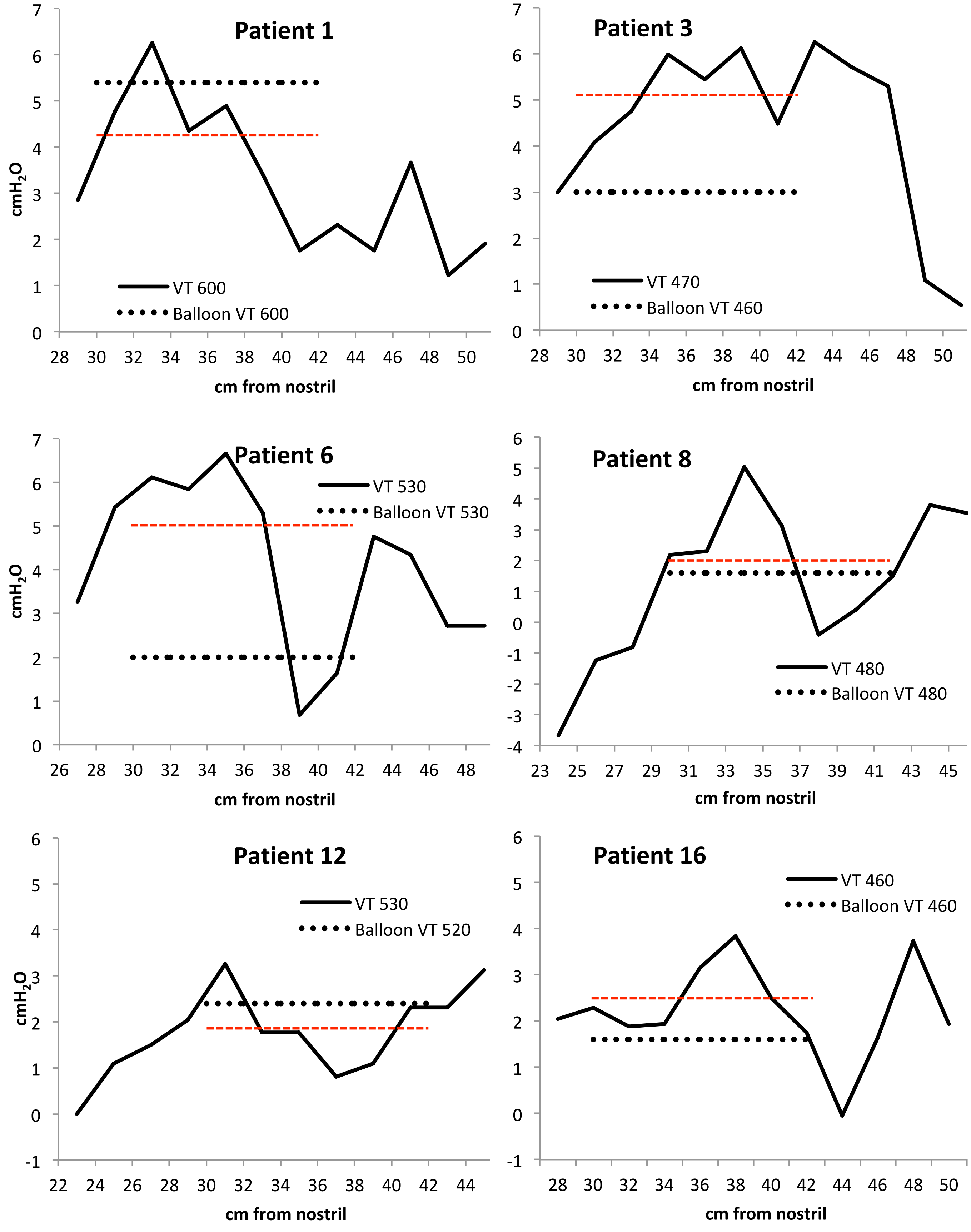

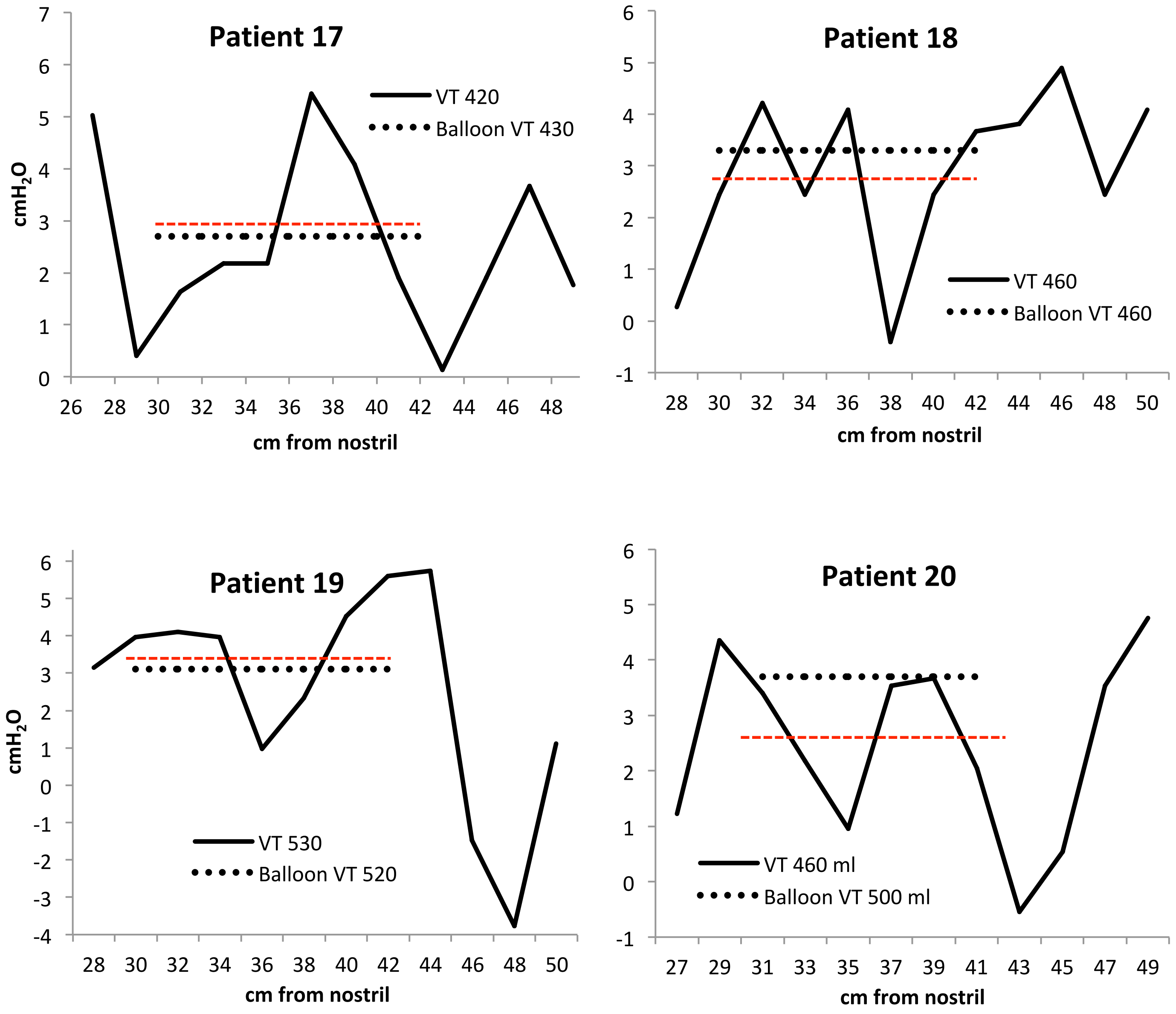
Patient 17
VT 420
Balloon VT 430
cmH2O
cm from nostril
Patient 18
VT 460
Balloon VT 460
cm from nostril
Patient 19
VT 530
Balloon VT 520
cmH2O
cm from nostril
Patient 20
VT 460 ml
Balloon VT 500 ml
cm from nostril

Supplementary Table 3
Comparison of esophageal pressure measured with HRM and conventional balloon catheter

	Ventilator settings	Mean difference	95% Confidence interval
End-expiratory esophageal pressure	Mean PEEP 10.4 cmH_2O	3.7 cmH_2O	2.1-5.3 cmH_2O
Tidal variations in esophageal pressure	VT≈6 ml/kg IBW at baseline PEEP	1.3 cmH_2O	0.1-2.5 cmH_2O

Supplementary Figure 10
Comparison of end-expiratory esophageal pressure measured with HRM and conventional balloon catheter according to Bland and Altman

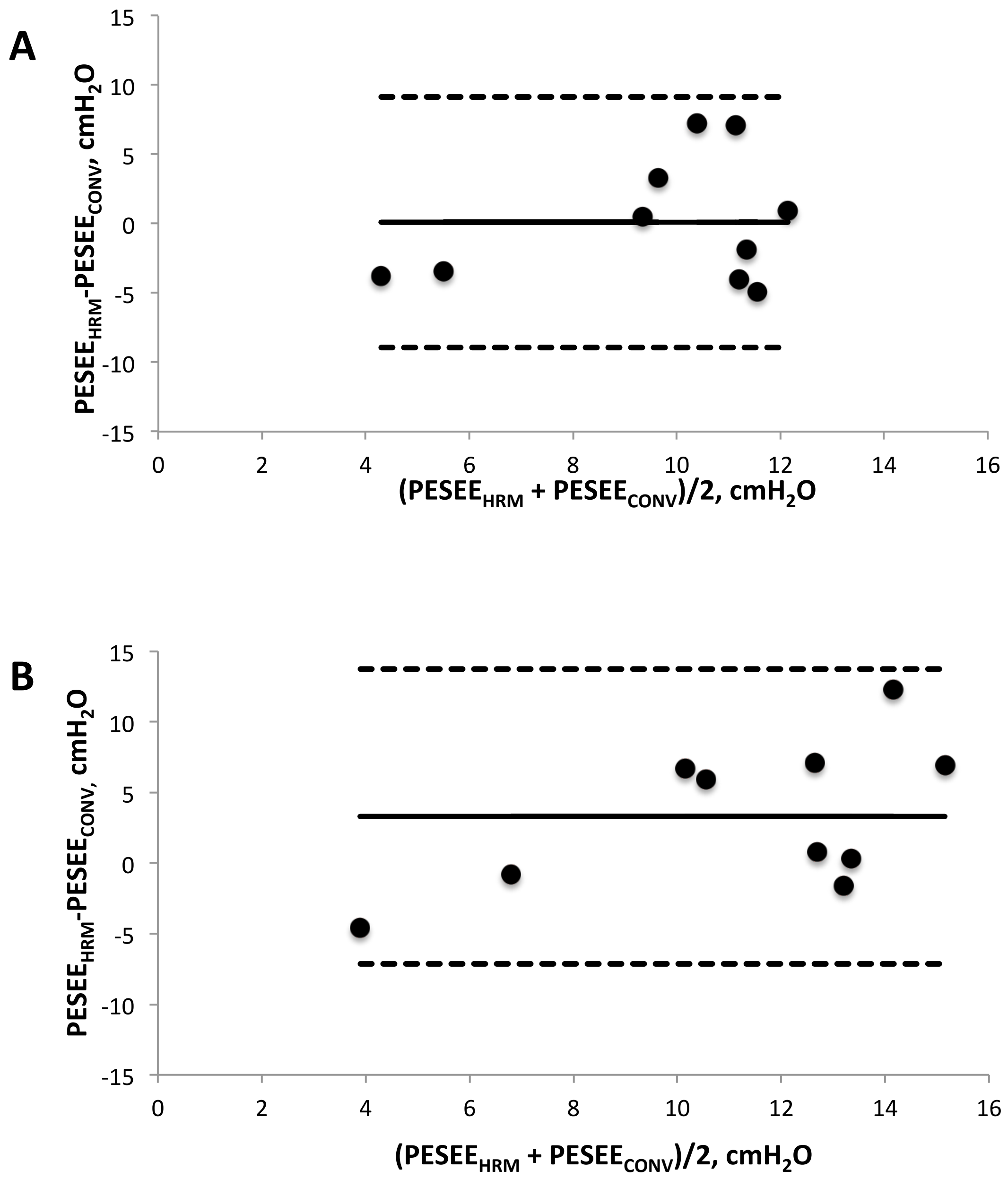

Supplementary Figure 11
Positive pressure occlusion test:
Change in esophageal pressure during chest compression

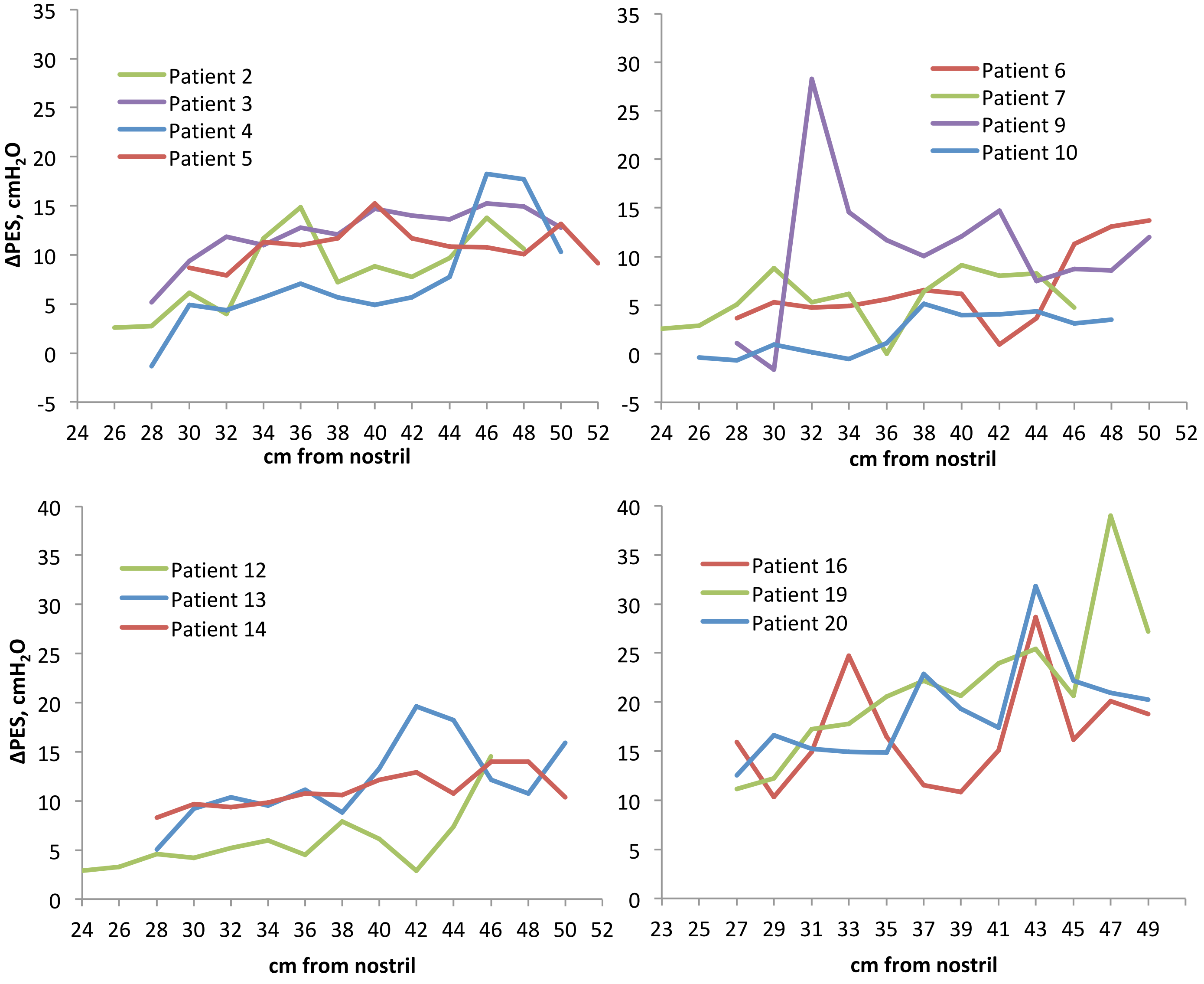

Supplementary Figure 12
End-expiratory esophageal pressure:
comparison between sitting and supine positions

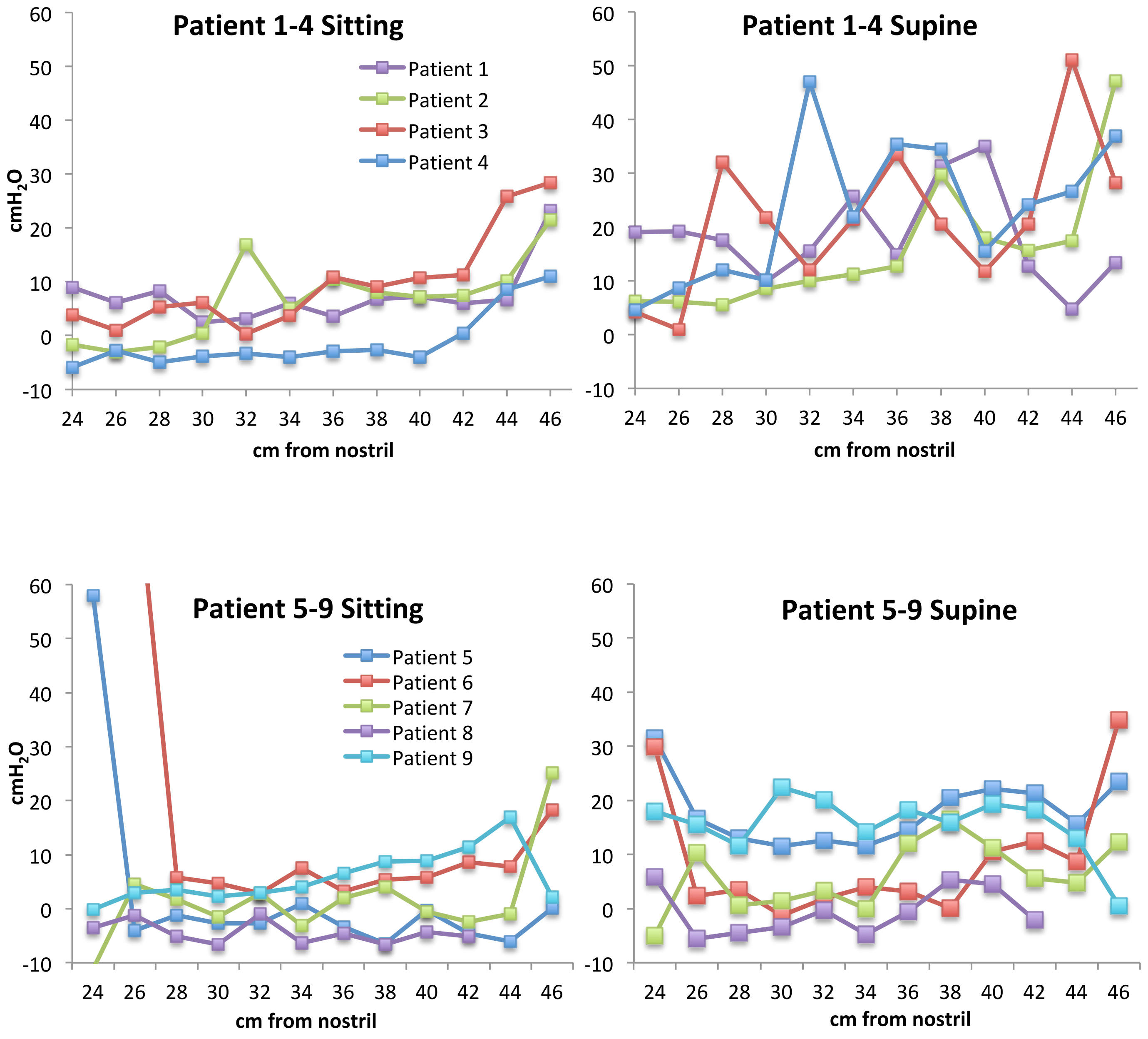

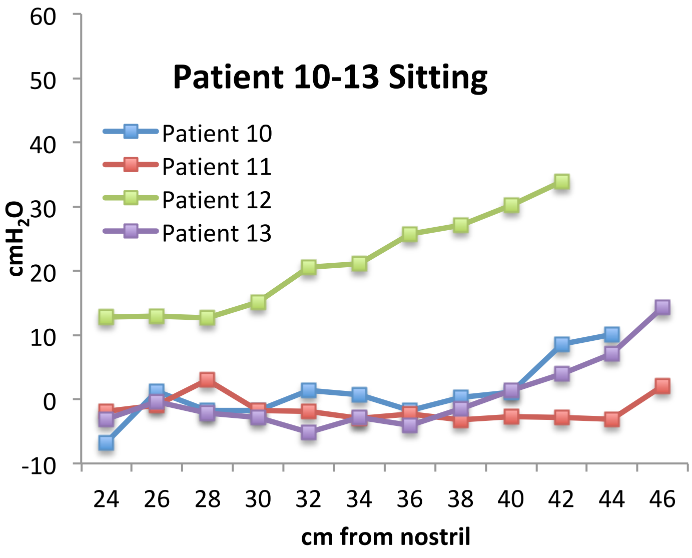
Patient 10-13 Sitting
Patient 10
Patient 11
Patient 12
Patient 13
cmH_2O
cm from nostril

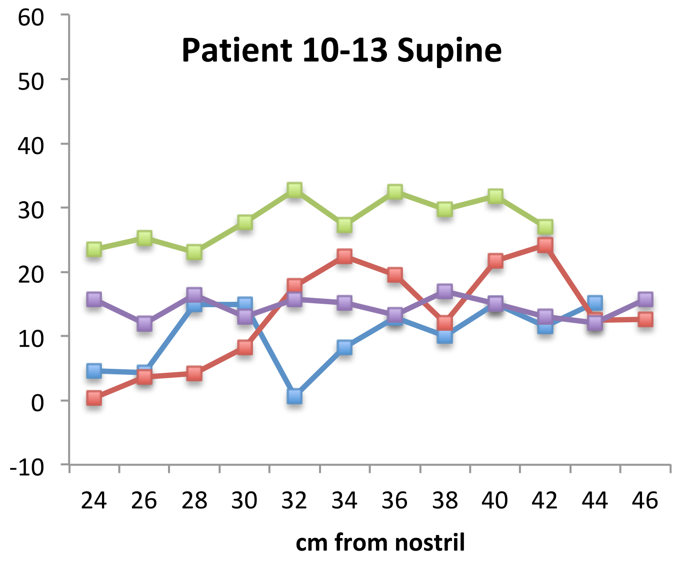
Patient 10-13 Supine
cm from nostril

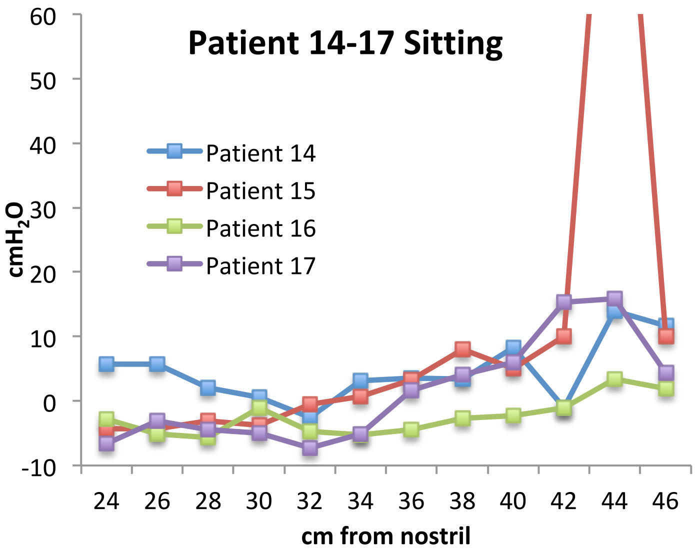
Patient 14-17 Sitting
Patient 14
Patient 15
Patient 16
Patient 17
cmH_2O
cm from nostril

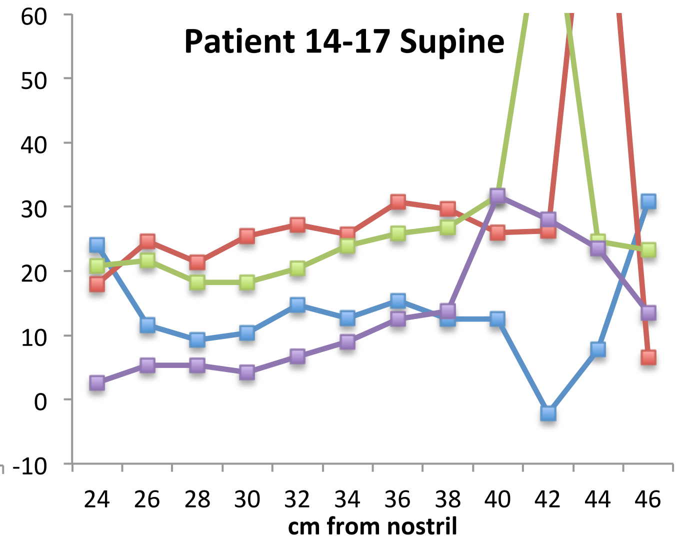
Patient 14-17 Supine
cm from nostril

Supplementary Figure 13
Effect of body position on esophageal pressure

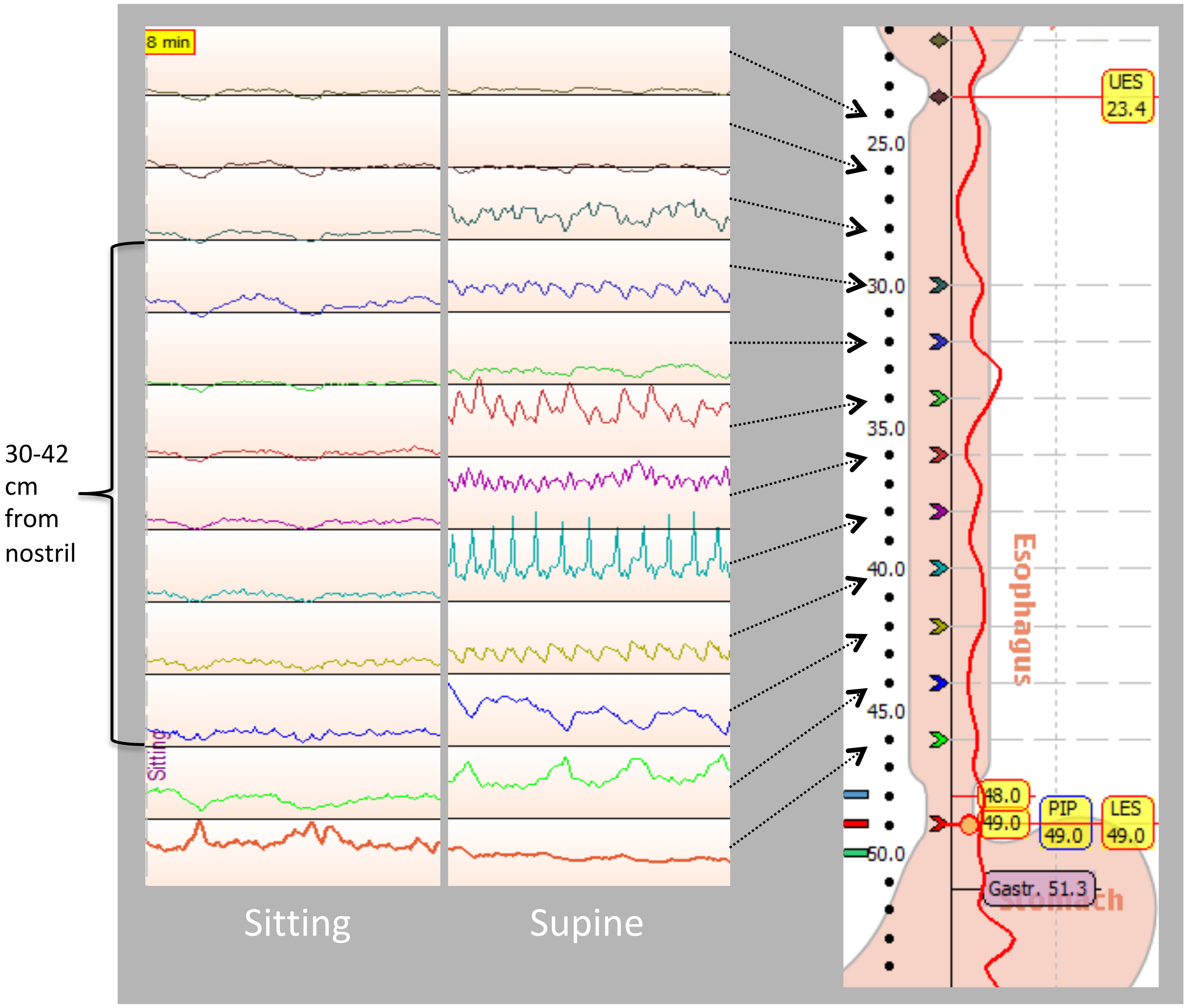

Supplement: Supplementary file 1 — Table S1. M = male, F = female, ICU = intensive care unit, OR = operating room. TBI = traumatic brain injury, ICB = intracerebral haemorrhage, SAH = subarachnoid haemorrhage. PaO2 measured in mmHg. LH = lung-healthy. In patient 5, information about length is missing. Figures presented as n (%) or mean (SD). Figure S1. Oesophageal pressures measured with high-resolution manometry. Right panel: an illustration of the oesophagus with a red line representing the pressure measured 1 cm apart at a chosen time. Number and dots next to the oesophagus represent cm from the nostril. UES = upper oesophageal sphincter. LES = lower oesophageal sphincter. Left panel: pressures measured 2 cm apart along the oesophagus 24–46 cm from the nostril. Number on the left represents cm from the nostril, and bold numbers next to pressure curves is the pressure (in mmHg) measured at that level at a chosen time. Line representing the chosen time is not visible in the figure. Figure S2. Individual curves for each patient showing end-expiratory oesophageal pressure along the oesophagus at different PEEP levels. Measurements performed with the high-resolution manometry catheter. Table S2.Intra-individual variation in oesophageal pressure described with coefficient of variation, calculated as within-subject standard deviation divided by the mean (SD/mean). Values in the table represent the mean and median from all patients. Coefficient of variation is presented for end-expiratory pressures at different PEEP levels and for tidal variations in oesophageal pressure at different tidal volumes. 22 cm of oesophagus (ESOTOT) and oesophagus 30–42 cm from the nostril (ESOLOW) are presented separately, see the “Methods” section. Figure S3. Figure shows how an increase of PEEP effects oesophageal pressure in two representative patients. When PEEP is increased, cardiac oscillations decrease which at some levels causes oesophageal pressure to decrease, black arrows. At other levels the oesophageal pressure in [file 13054_2019_2484_MOESM1_ESM.pdf]
